# Extracellular Vesicles Enriched with Moonlighting Metalloproteinase Are Highly Transmissive, Pro-Tumorigenic, and Trans-Activates Cellular Communication Network Factor (*CCN2/CTGF*): CRISPR against Cancer

**DOI:** 10.3390/cancers12040881

**Published:** 2020-04-04

**Authors:** Yuka Okusha, Takanori Eguchi, Manh T. Tran, Chiharu Sogawa, Kaya Yoshida, Mami Itagaki, Eman A. Taha, Kisho Ono, Eriko Aoyama, Hirohiko Okamura, Ken-ichi Kozaki, Stuart K. Calderwood, Masaharu Takigawa, Kuniaki Okamoto

**Affiliations:** 1 Department of Dental Pharmacology, Graduate School of Medicine, Dentistry and Pharmaceutical Sciences, Okayama University, Okayama 700-8525, Japan; yokusha@bidmc.harvard.edu (Y.O.); trantienmanh1508@gmail.com (M.T.T.); caoki@md.okayama-u.ac.jp (C.S.); mami1515@s.okayama-u.ac.jp (M.I.); pj7l8pfb@s.okayama-u.ac.jp (E.A.T.); ken-1@okayama-u.ac.jp (K.-i.K.); k-oka@okayama-u.ac.jp (K.O.); 2 Division of Molecular and Cellular Biology, Department of Radiation Oncology, Beth Israel Deaconess Medical Center, Harvard Medical School, Boston, MA 02115, USA; scalderw@bidmc.harvard.edu; 3 Advanced Research Center for Oral and Craniofacial Sciences, Graduate School of Medicine, Dentistry and Pharmaceutical Sciences, Okayama University, Okayama 700-8525, Japan; eaoyama@cc.okayama-u.ac.jp (E.A.); takigawa@md.okayama-u.ac.jp (M.T.); 4 Department of Oral Healthcare Education, Institute of Biomedical Sciences, Tokushima University Graduate School, Tokushima 770-8504, Japan; kaya@tokushima-u.ac.jp; 5 Research program for undergraduate students, Okayama University Dental School, Okayama 700-8525, Japan; 6 Department of Medical Bioengineering, Graduate School of Natural Science and Technology, Okayama University, Okayama 700-8530, Japan; 7 Department of Biochemistry, Ain Shams University Faculty of Science, Cairo 11566, Egypt; 8 Department of Oral and Maxillofacial Surgery, Okayama University Hospital, Okayama 700-0914, Japan; de20012@s.okayama-u.ac.jp; 9 Department of Oral Morphology, Dentistry and Pharmaceutical Sciences, Okayama University Graduate School of Medicine, Okayama 700-8525, Japan; hiro-okamura@okayama-u.ac.jp

**Keywords:** matrix metalloproteinase, moonlighting metalloproteinase (MMP), protein moonlighting, transcription factor, extracellular vesicles, oncosome, genome editing, CRISPR, cellular communication network factor, CCN2/CTGF

## Abstract

Matrix metalloproteinase 3 (MMP3) plays multiple roles in extracellular proteolysis as well as intracellular transcription, prompting a new definition of moonlighting metalloproteinase (MMP), according to a definition of protein moonlighting (or gene sharing), a phenomenon by which a protein can perform more than one function. Indeed, connective tissue growth factor (CTGF, aka cellular communication network factor 2 (CCN2)) is transcriptionally induced as well as cleaved by MMP3. Moreover, several members of the MMP family have been found within tumor-derived extracellular vesicles (EVs). We here investigated the roles of MMP3-rich EVs in tumor progression, molecular transmission, and gene regulation. EVs derived from a rapidly metastatic cancer cell line (LuM1) were enriched in MMP3 and a C-terminal half fragment of CCN2/CTGF. MMP3-rich, LuM1-derived EVs were disseminated to multiple organs through body fluid and were pro-tumorigenic in an allograft mouse model, which prompted us to define LuM1-EVs as oncosomes in the present study. Oncosome-derived MMP3 was transferred into recipient cell nuclei and thereby trans-activated the *CCN2/CTGF* promoter, and induced CCN2/CTGF production in vitro. TRENDIC and other cis-elements in the *CCN2/CTGF* promoter were essential for the oncosomal responsivity. The CRISPR/Cas9-mediated knockout of MMP3 showed significant anti-tumor effects such as the inhibition of migration and invasion of tumor cells, and a reduction in CCN2/CTGF promoter activity and fragmentations in vitro. A high expression level of MMP3 or CCN2/CTGF mRNA was prognostic and unfavorable in particular types of cancers including head and neck, lung, pancreatic, cervical, stomach, and urothelial cancers. These data newly demonstrate that oncogenic EVs-derived MMP is a transmissive trans-activator for the cellular communication network gene and promotes tumorigenesis at distant sites.

## 1. Introduction

Cancer is one of the most common causes of death, with its lethality involving metastasis and therapy resistance. Poor prognosis in cancer patients is associated with rapid tumor progression, a permissive tumor–stroma interactive microenvironment, and the dissemination of tumor cells to blood circulation and distant organs where metastatic secondary tumors are formed [1,2]. Recent studies have shown that extracellular vesicles (EVs) released from the cells can transfer bioactive molecules to neighboring cells and deliver them to distant organs through body fluids such as the bloodstream. EV-mediated molecular transfer is essential for several events in tumor progression, including epithelial–mesenchymal transition (EMT), tumor–stroma interaction, and metastasis [3,4,5]. EVs can promote the dissemination of cancer cells through body fluids [6], the formation of pre-/pro-metastatic niche [7], and the education of myeloid cells [8]. Tumor EVs often contain oncogenic proteins such as EGFRvIII, EGFR, heat shock proteins (HSP), CD326/EpCAM, and KIT [3,4,9,10,11,12,13,14,15]. It has been shown that the molecular transfer of oncogenic proteins can transform recipient cells, inducing phenotypes such as EMT [3,9], which prompted the use of the conceptual term “oncosome” for oncogenic vesicles. Previous studies have reported oncosomes (100–400 nm; containing EGFRvIII, released by glioblastoma) and large oncosomes (LO: >1,000 nm to >10,000 nm, released by metastatic prostate cancer cells) [16]. These terms are not synonymous or interchangeable, because they have different origins, conceptual contexts, EV size references, and contents, and were introduced at different times. Notably, many EV proteins are in common with protein secretome, including matrix metalloproteinases (MMP) and cellular communication network factors (CCN family proteins) [17]. In the present study, we characterize tumor-derived EVs, by pro-tumorigenic and transmissive potentials, size, and a relevant oncogenic cargo. In other words, we aimed to investigate a mechanism by which vesicular MMP3 promotes tumor progression in vitro and in vivo.

It has been shown that MMP3 and CCN2 (aka connective tissue growth factor (CTGF)) are often increased in tumor–stroma tissues or patients’ serum, and are thus biomarkers correlated with poor prognosis in cancer [18,19,20,21,22]. It has been also shown that MMP3 and CCN2/CTGF promote tumor progression through processes including rapid metastasis and tumor–stroma interactions [23,24,25,26]. CCN2/CTGF up-regulates MMP family proteins in cancer [27], while MMP3 regulates CCN2/CTGF by two mechanisms, including (i) intracellular MMP3, which directly activates the *CCN2/CTGF* gene to induce the production of CCN2/CTGF protein in chondrocytes [28,29] and (ii) MMPs, which cleave CCN2/CTGF to generate bioactive fragments essential for angiogenesis and osteoclastogenesis [30,31]. Indeed, previously we characterized the mechanisms underlying the robust expression of CCN2/CTGF in connective tissues such as cartilage and tumors [32,33]. Promoter analysis of *CCN2/CTGF* revealed a *cis*-element designated as a transcriptional enhancer dominant in chondrocytes (TRENDIC) [34]. One of the TRENDIC-binding proteins was identified as MMP3. MMP3 overexpression enhanced *CCN2/CTGF* promoter activity in the human chondrosarcoma-derived chondrocytic cell line HCS-2/8 and non-basal type, triple-negative breast cancer cell line MDA-MB-231 [28]. The intranuclear translocation of recombinant MMP3, as well as endogenous MMP3, was observed under confocal laser scanning microscopy (CLSM) [28]. The DNA-binding of MMP3 was next demonstrated by gel shift and chromatin immunoprecipitation assays. Human MMP3 also contains six basic amino acid clusters, which were shown to be nuclear localization signals (NLS) [29]. An MMP3-specific inhibitor inhibited the activity of the *CCN2/CTGF* promoter, suggesting that MMP3 proteolytic activity was required for the transcriptional role of this enzyme, although the MMP2/9 inhibitor was ineffective in HSC-2/8 cells [28]. Nuclear MMP3 immunostaining was pronounced in cartilage tissues in the normal and arthritic mouse model. Therefore, according to the definition of protein moonlighting (or gene sharing) as a phenomenon by which a protein can perform more than one function, we re-define MMP3 as a moonlighting metalloproteinase (MMP) that is a proteolytic enzyme as well as a transcription factor. However, the vesicle-associated transport and roles of MMP3 in cancer have not previously been unveiled, mechanisms that we aimed to investigate here.

We have recently developed an allograftable metastatic tumor model in mice [23,35]. A colon cancer cell line, Colon26, was generated from BALB/c mice and a rapidly metastatic cancer cell line, LuM1, was further generated by the inoculation of Colon26 into mice and repetitive metastasis experiments [36]. Notably, the LuM1 cells expressed MMP3 and MMP9 at high levels compared to Colon26. RNA interference (RNAi) targeting MMP3 and/or MMP9 significantly lowered tumor growth and metastasis in the allograft mouse model [23]. In the present study, we generated MMP3-knockout (MMP3-KO) cells, by using clustered, regularly interspaced, short palindromic repeat (CRISPR)-Cas9 genome editing technology on the LuM1 cells and compared the oncogenic effects of these cells and their EVs. We also have shown that MMP3 was distributed at the tumor–stroma border area and immunostained in cell nuclei in the tumor allograft mouse model [23]. We therefore hypothesized that the molecular transmission of MMP3 during tumor–stroma interaction could underlie the pro-tumorigenic and pro-metastatic roles of this multi-functional proteinase, and we also aimed to investigate this mechanism in the present study.

## 2. Results

### 2.1. Metastatic Tumor-Derived, MMP3-Rich EVs Are Highly Transmissive

**Figure 1:**
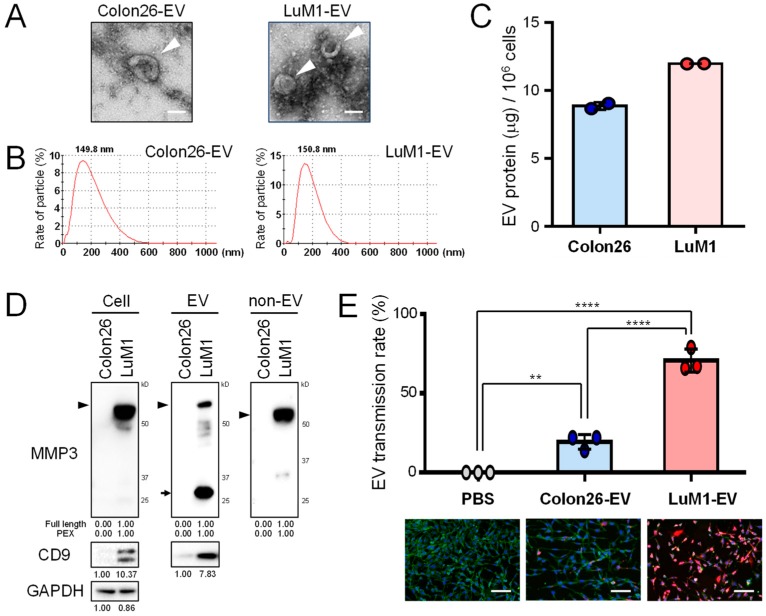
Metastatic tumor-derived, matrix metalloproteinase 3 (MMP3)-rich extracellular vesicles (EVs) are highly transmissive. The roles of EVs derived from low-metastatic Colon26 cells vs. high-metastatic LuM1 cells were compared. (**A**) Representative TEM images of EVs. Arrowheads indicate EVs with cup-shaped morphology. Scale bars, 100 nm. (**B**) Particle diameter distribution of EV fractions. (**C**) EV protein release from Colon26 vs LuM1 cells. EV protein concentrations (μg per 10^6^ cells) were shown. (**D**) Western blot analysis of MMP3 and CD9 in cell lysates, EV and non-EV fractions. Arrowhead indicates full-length MMP3 (54 kD). Arrows indicate the 25-kD PEX isoform of MMP3. For MMP3, the protein amount equivalent to 8 × 10^4^ cells (Colon26, 0.72 μg; LuM1, 0.96 μg) was loaded to each lane. For CD9 and GAPDH, the protein amount equivalent to 1.2 × 10^5^ cells (Colon26, 1.09 μg; LuM1, 1.44 μg) was loaded to each lane. (**E**) Transmission efficiencies of Colon26-EVs vs LuM1-EVs. EVs were labeled with red fluorescent ceramide and added to culture media of recipient Colon26 cells at a final concentration of 11.5 μg/mL for 24 h. Cells were fixed and stained with ActinGreen and DAPI. Top, EV transmission efficiencies. The efficiencies were the ratio of transmitted EV-positive cells to the total number of cells. ** *p* < 0.01, **** *p* < 0.0001, n = 3 fields. Bottom, representative images of EV transmission. Scale bars, 100 μm. The experiments were repeated twice in Figure 1C–E.

To characterize highly metastatic, LuM1-derived EVs, we first prepared EVs from the culture supernatants of Colon26 and LuM1 cells and then analyzed their morphologies, and physical characteristics as well as their transmissive and pro-tumorigenic potentials. Vesicular structures with a cup-shaped morphology sized between 50 and 200 nm were found in EV fractions from both LuM1 and Colon26 cells under transmission electron microscopy (TEM) (Figure 1A). Particle diameter distribution analysis revealed that both cell types secreted EVs (50–400 nm) with single peaks at approximately 150 nm (Figure 1B). LuM1 tended to release more EV proteins than Colon26 cells (Figure 1C), consistent with our previous report [35].

Next, we examined whether MMP3 was contained in the EVs and non-vesicular extracellular fractions of LuM1 and Colon26 cells. The anti-MMP3 C-terminus-specific antibody detected full-length MMP3 at markedly higher levels in the cell lysate, EV fraction and non-EV fraction of LuM1 cells (Figure 1D, arrowheads; Figure S1). Notably, the same anti-MMP3 antibody detected multiple fragments of MMP3 in cellular, EV, and non-EV fractions, including the C-terminal PEX fragment of MMP3 in the LuM1-EV fraction, suggesting that matured active metalloproteinases could self-cleave MMP3 in LuM1 cells and on EVs (Figure 1D, arrows). 

Many EV markers have been up-listed in the MISEV2018 guideline [37], including CD9 (a category-1 EV marker) [37], HSP90α/HSP90AA1 (a stressome marker) [11,12,13,14,15], β-actin (a category-2b EV marker), and secretory proteins recovered with EVs as category-5 EV markers such as growth factors, ECM, and MMPs. In our study, CD9 was detected in LuM1-EVs and the cell lysate at higher levels than in Colon26, suggesting that LuM1 produced CD9/MMP3-EVs while Colon26 incorporated other protein types within vesicles (Figure 1D). In contrast, HSP90α was detected in Colon26-EVs and the cell lysate at higher levels than in LuM1, suggesting that LuM1 and Colon26 released different secretomes (data not shown) [38]. Beta-actin was detected in both Colon26-EVs and LuM1-EVs (data not shown) [38]. These data indicated that LuM1 cells released CD9/MMP3-EVs while Colon26 released other protein types with vesicles.

Next, we examined whether EV transmissive potentials could be different between LuM1- and Colon26-derived EVs. LuM1-EVs were more actively transmitted into Colon26 recipient cells compared to Colon26-EVs (Figure 1E), suggesting that LuM1-EVs may promote more endosomal escape than Colon26-EVs. Furthermore, the MMP3 levels in recipient Colon26 cells were markedly increased after the addition of MMP3-rich LuM1-EVs (Figure S2; see later data of molecular transfer).

These experiments suggested that MMP3-rich EVs released by the more aggressive cancer cells were highly transmissive into recipient cells. We also propose that the highly transmissive potential of EVs could be a property of oncosomes, to be defined in the present study.

### 2.2. The Pro-Tumorigenic Effects of LuM1-EVs In Vivo

**Figure 2:**
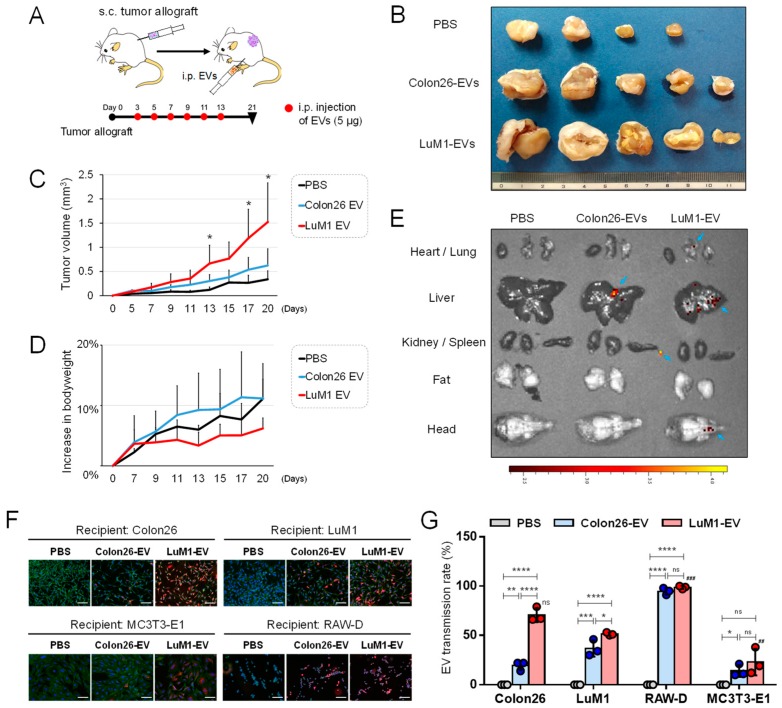
Pro-tumorigenic effects of oncosomes mediated through body fluid. (**A**) A schema of the animal experiment. Colon26 cells were injected subcutaneously (s.c.) into the side abdominal wall of BALB/c mice at the volume of 5.0 × 10^5^ cells solved in 0.5 ml PBS. After the allograft, Colon26-EV fraction, LuM1-EV fraction or PBS were injected intraperitoneally (i.p.) at the volume of 5 µg of EVs solved in 0.5 ml PBS from day 3 to day 13, every other day, 6 times. (**B**) Representative images of subcutaneous tumors on day 21 after the i.p. (**C**) Tumor growth and (**D**) bodyweights altered by exogenous EVs. PBS group, n = 3; Colon26 EV group, n = 4; LuM1 EV group, n = 5. Mean + SD, * *p* < 0.05 compared to the PBS group. The percentage increases in bodyweights to day 0 were plotted. (**E**) In vivo imaging of EV transmission. EVs were labeled with Cy7 and then i.p. injected. In vivo images were taken 24 h after the administration. (**F**,**G**) Transmissive efficiencies of Colon26- or LuM1-EVs into various recipient cells (Colon26, LuM1, macrophage-like RAW-D, and calvaria-derived MC3T3-E1). EVs were labeled with red fluorescent ceramide and added to culture media of Colon26, LuM1, MC3T3-E1, or RAW-D recipient cells at a final concentration of 11.5 μg/mL for 24 h. PBS, a negative control. Cells were fixed and stained with ActinGreen and DAPI. (**F**) Representative images of recipient cells. (**G**) EV transmission rate. The rates were calculated as the ratio of transmitted fluor EV-positive cells to the total number of cells. * *p* < 0.05, ** *p* = 0.0073, *** *p* = 0.0002, **** *p* < 0.0001, ### *p* = 0.0003 (LuM1 vs. RAW-D recipients), ## *p* = 0.0087 (LuM1 vs. MC3T3-E1 recipients), n = 3 fields. ns, not significant. The data of Colon26 were also shown in Figure 1. The experiments were repeated twice each in Figure 2A–D.

We have seen that LuM1 cells were much more tumorigenic and metastatic than Colon26 in the allograft model [23,35]. Then, we hypothesized that LuM1-EVs could have more protumorigenic potential than Colon26-EVs. To investigate the body fluid-mediated dissemination and pro-tumorigenic effect of EVs, we next examined whether intraperitoneally injected LuM1-EVs altered subcutaneously allografted Colon26-tumors in mice. Indeed, LuM1-EVs significantly promoted tumor growth of Colon26 compared to the PBS-injected control group and the Colon26-EV group (Figure 2A–C), suggesting that LuM1-EVs disseminated through body fluid and promoted tumorigenesis. 

The body weight of mice increased more rapidly in the tumor/Colon26-EV injection group compared to the tumor/PBS-injected group. On the other hand, body weight loss in mice was seen after the injection of the LuM1-EVs, suggesting that cachexia might be caused by LuM1-derived EVs (Figure 2D). To monitor the organ-tropic (tissue-specific) transmission of EVs, we intraperitoneally injected Cy7-labeled EVs into mice and then visualized the targeting of the EVs using an in vivo imaging system (IVIS). LuM1-EVs distributed to the lung, where Colon26-EVs were undetectable (Figure 2E), consistent with the lung-tropic metastatic property of LuM1 cells [23]. LuM1-EVs also distributed to the head, where Colon26-EVs were undetectable. However, both LuM1-EVs and Colon26-EVs primarily distributed to the livers.

To investigate tissue or cell-type specificities targeted by LuM1-EVs, we examined transmission efficiencies of LuM1-EVs into various recipient cells such as LuM1 cells themselves, the lower grade tumor cells (Colon26), mouse macrophage-like cell line (RAW-D), and mouse calvaria-derived osteoblastic cell line (MC3T3-E1). LuM1-EVs were more efficiently transmitted into the tumor cells (both the lower grade Colon26 and LuM1) than Colon26-EVs (Figure 2F,G), suggesting that the transmissive and protumorigenic properties of EVs in tumors are novel criteria of the oncosomes which we aim to define in the current study, consistent with the data shown in Figure 2C. LuM1-EVs and Colon26-EVs were similarly most efficiently taken up by macrophage-like RAW-D cells among the four types of cells tested. These findings suggest that macrophages could robustly take up EVs and then potentially deliver some EV cargos to the liver and/or tumors, but are not involved in the different protumorigenic potentials between Colon26-EVs versus LuM1-EVs. LuM1-EVs and Colon26-EVs were also transmissive to MC3T3-E1, although the effect was less compared to other types of cells. Before these experiments, we had two mechanistic hypotheses, including that (i) LuM1-EVs were efficiently transmissive through body fluid and tissues and better taken up by Colon26-tumors than Colon26-EVs and/or (ii) macrophages efficiently took up EVs and then delivered them to tumors. LuM1-EVs were much more efficiently transmitted into Colon26 than Colon26-EVs, while EV uptake efficiencies by macrophages were not different between Colon26-EVs and LuM1-EVs. This is a current rationale of the LuM1-derived EVs targeting to Colon26 to promote tumorigenicity. 

These data indicate that the MMP3-rich EVs (oncosomes) released by the aggressive cancer cells are highly transmissive to multiple organs including the lung, head, and liver, and pro-tumorigenic through the body fluids.

### 2.3. Establishment of MMP3 Knockout Cells Using the CRISPR/Cas9 Genome Editing Technology

To establish MMP3-knockout cells, we next edited the genome of LuM1 cells using the CRISPR/Cas9 system. We found a targetable repetitive CRISPR sequence (5′-TGCA……TGCA-3′) followed by PAM sequences (5′-TGG -3′, 5′-AGG -3′, or 5′-GGG -3′) in exon 1 and exon 2 in *Mmp3* gene in murine chromosome 9 (position 7445958). Among the many candidates, we designed and synthesized guide RNA (gRNA) #1 and #2 (Table 1) targeting the CRISPR sequence in exon 1 and exon 2, respectively, of the *Mmp3* gene without any mismatch, but with 2-3 or more nucleotide mismatches on other chromosomal sequences to minimize off-target effects. We finally used the gRNA #1 with a higher cleavage efficiency than gRNA #2 and transfected a ribonucleoprotein (RNP) complex composed of the gRNA and recombinant Cas9 (rCas9) nuclease using a transfection reagent.

**Figure 3:**
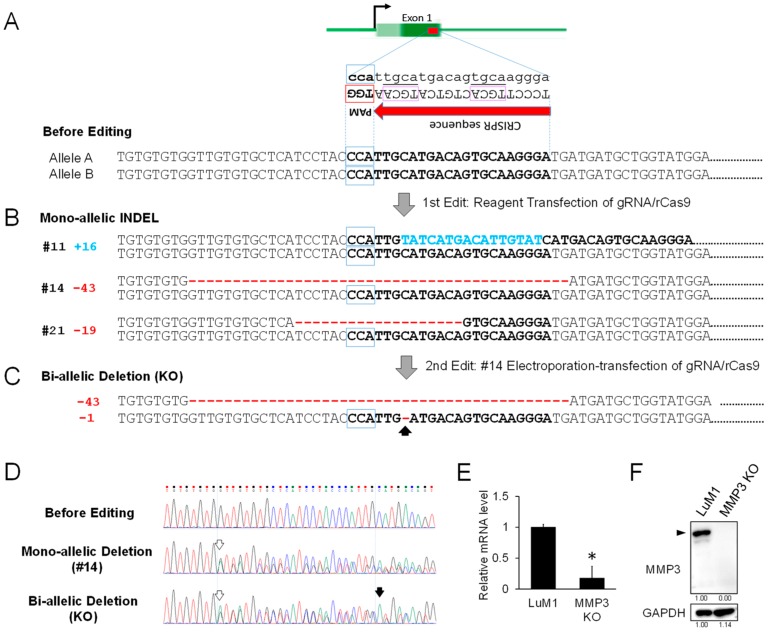
Establishment of MMP3-KO cells using CRISPR/Cas9 genome editing technology. (**A**) Schemes representing the targeting of exon 1 in the *Mmp3* gene. Top, a scheme of *Mmp3* gene. Dark green box, a coding region of exon 1 including the target sequence. Bright green box, 5′-untranslated region. Middle, a CRISPR sequence containing 5′-TGCA-3′ repeat enclosed with purple square, tailed with PAM sequence (TGG) in the antisense strand. Bottom, a partial sequence of *Mmp3* exon 1 including the target sequence (shown in bold). (**B**) The sequences of mono-allelic INDEL clones. Three types of mono-allelic INDEL clones were obtained. Clone #11 contained a 16-bp insertion sequence, shown in blue. Clones #14 and #21 conferred 43-bp and 19-bp deletions, respectively, shown in red. (**C**) DNA sequences of the bi-allelic deletion clone. This clone was obtained from the clone #14 with an additional single nucleotide deletion in the counterpart allele, as indicated by an arrow. (**D**) DNA sequences before editing, of mono-allelic deletion (#14 clone) and bi-allelic deletion (KO). White arrows, the position of the first deletion. Black arrow, the position of the single nucleotide deletion in the 2nd edit. (**E**) RT-qPCR analysis of *Mmp3* mRNA in the parental LuM1 cells and the MMP3-KO cells. The mRNA level of *Mmp3* was decreased while the mutant mRNA of *Mmp3* was detectable. The relative mRNA levels were normalized to *Gapdh*, an internal control. * *p* < 0.05, n = 6. (**F**) Western blot showing MMP3 in the cell lysates. Arrowhead indicates full-length MMP3 (54 kD). The experiments were repeated thrice in Figure 3E,F.

We next established three clones of mono-allelic insertion or deletion (INDEL) in a total of 21 clones (Figure 3B, Table 2, Figure S3). The three types of mono-allelic INDEL clones were composed of two heterogenic deletion clones (clone #14 with 43-base deletion and clone #21 with 19-base deletion) and one heterogenic insertion clone (clone #11 with 16-base insertion).

To establish a bi-allelic deletion clone (MMP3-KO), we secondly transfected the same RNP complex into clone #14 using an electroporation-transfection method. By single-cell cloning, we established eight clones, then found a bi-allelic deletion clone with additional single nucleotide deletion with 75% efficiency in the counterpart allele (Figure 3C,D; Table 2), suggesting that these bi-allelic deletions cause frame-shifts in exon 1 and the subsequent premature termination codon. To verify the knockout of MMP3, we then performed RT-qPCR and Western blotting. The mRNA levels of MMP3 in the bi-allelic deletion clone was significantly lower than those of LuM1 cells, suggesting that the mutant mRNAs of MMP3 were barely transcribed and unstable (Figure 3E). MMP3 protein was undetectable in the MMP3-KO cells while being readily detectable in the parental LuM1 cells (Figure 3F).

### 2.4. The Anti-Tumor Effect of MMP3-Knockout

**Figure 4:**
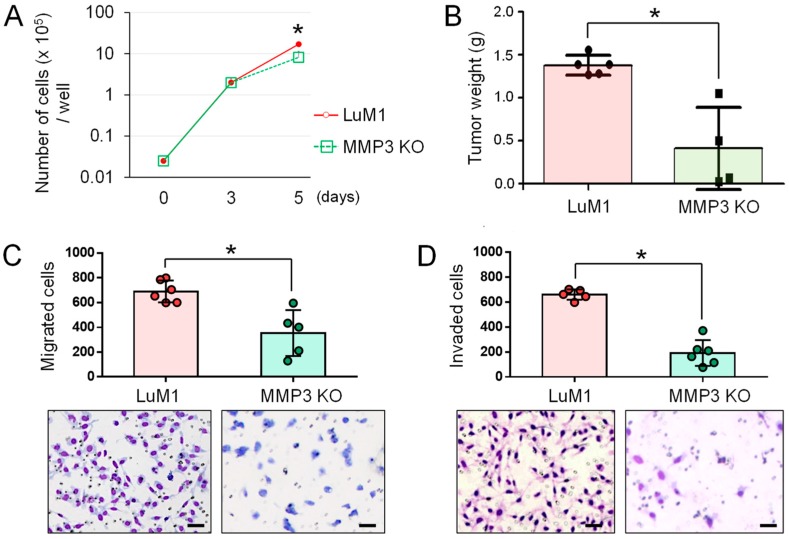
The knockout of MMP3 suppressed tumor progression. (**A**) Growth curves of LuM1 and MMP3-KO cells. n = 3. * *p* < 0.05. (**B**) Subcutaneous tumor weights of LuM1 vs. MMP3-KO cells in the allograft model. Tumor weights were measured on day 21 after transplantation. LuM1 group, n = 6; MMP3 KO group, n = 5. * *p* < 0.05. (**C**) Column scatterplot of in vitro migration activities altered by MMP3 knockout. Top, the number of migrated cells. n = 5, * *p* < 0.05. Bottom, representative images of migrated cells stained with Diff-Quick. Scale bars, 50 µm. (**D**) Column scatterplot of in vitro invasion activities. Top, the number of invaded cells. n = 5, * *p* < 0.05. Bottom, representative images of invaded cells stained with Diff-Quick. Scale bars, 50 µm. The experiments were repeated twice in Figure 4A,B and thrice in Figure 4C,D.

To elucidate the potential role of MMP3-KO in tumor progression, we examined whether the knockout of MMP3 altered the proliferation, migration, and invasion in vitro and tumor growth in vivo. MMP3-KO cells grew more slowly in vitro by Day 5 compared to LuM1 cells (Figure 4A). The knockout of MMP3 significantly inhibited tumor growth in mice in the allograft model (Figure 4B). Cellular migration and invasion were also significantly inhibited by the deletion of MMP3 compared to the MMP3-high LuM1 cells (Figure 4C,D). 

These data indicated that MMP3 plays a key role in tumor progression, processes including migration, invasion, and tumor growth, while MMP3-KO is an effective anti-tumor method targeting aggressive cancer cells.

### 2.5. MMP3 Positively Regulates the Expression and Secretion of CCN2/CTGF from the Metastatic Cancer Cells

**Figure 5:**
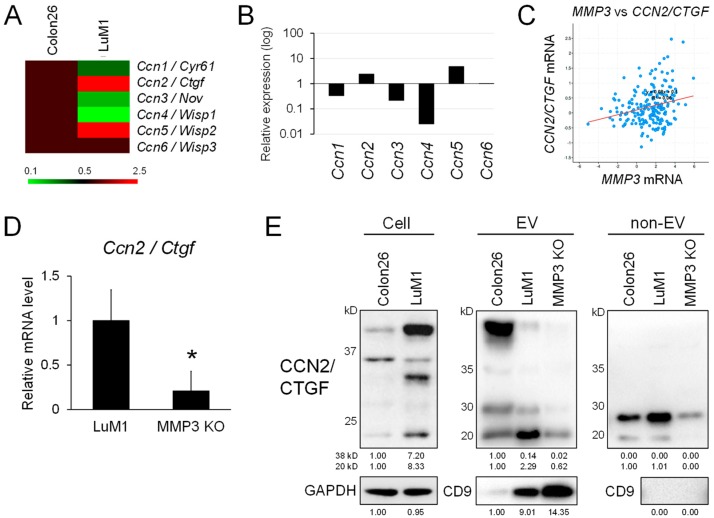
MMP3 knockout diminished gene expression and C-terminal fragments of CCN2/CTGF. (**A**–**C**) The positive correlation between MMP3 and CCN2/CTGF expression. (**A**) Heatmap analysis of the CCN gene family members between LuM1 vs. Colon26 cells. The data are from microarray analysis. (**B**) Relative mRNA expression levels of CCN gene family members in LuM1 vs. Colon26 cells. (**C**) Scatter plot analysis showing co-expression correlation between *MMP3* vs. *CCN2/CTGF* in patients-derived tumor samples of colorectal adenocarcinomas (632 cases). (**D**) RT-qPCR analysis of *Ccn2/Ctgf* transcripts expressed in LuM1 vs. MMP3-KO cells. n = 6, * *p* < 0.05. (**E**) Western blot analysis of CCN2/CTGF fragments in cellular, EV and non-EV fractions. C-terminal TSP1-CT fragments (20–25 kD) of CCN2/CTGF were at different levels among extracellular fractions of Colon26, LuM1, and MMP3-KO cells. CD9, a category-1 EV marker. GAPDH, loading control. The experiments were repeated twice in Figure 5D,E.

It has been shown that MMP3 positively regulates *CCN2/CTGF* gene expression in chondrocytes [28]. We therefore next examined whether the gene expression of cellular communication network gene family members composed of CCN1 to CCN6 was altered between Colon26 and LuM1 or by the targeted deletion of MMP3. Among the six CCN family members, the transcript levels of *Ccn2/Ctgf* and *Ccn5/Wisp2* were higher in LuM1 cells than those in Colon26 cells (Figure 5A,B). In contrast, the transcript levels of *Ccn1/Cyr61, Ccn3/Nov*, and *Ccn4/Wisp1* were diminished in LuM1 cells. 

We next examined the co-expression correlation between *MMP3* and *CCN2/CTGF* among 632 tumor samples derived from 632 colorectal adenocarcinoma patients registered in The Cancer Genome Atlas (TCGA). There was a marked co-expression correlation significance between *MMP3* and *CCN2/CTGF* (Figure 5C; y = 0.08x + 0.1, R^2^ = 0.06) in the colorectal adenocarcinoma cases, suggesting that MMP3 may positively regulate *CCN2/CTGF* expression in both human clinical tumors and mouse cancers. Consistently, the transcript level of *CCN2/CTGF* was significantly lowered in the MMP3-KO cells compared to LuM1 cells (Figure 5D). 

In addition to transcriptional regulation, MMPs are able to cleave CCN2/CTGF in the middle of the protein, separating N-terminal IGFBP-VWC modules and C-terminal TSP1-CT modules [30]. We detected the increased levels of C-terminal half fragments (approximately 20–25 kD, including the TSP1-CT modules) of CCN2/CTGF in LuM1 cells, their EV and non-EV fractions at higher levels compared to Colon26 cells (Figure 5E, Figure S4). The knockout of MMP3 reduced the CCN2/CTGF fragment levels in the extracellular fractions. These data suggested that MMP3 exerted at least two functions, including the up-regulation of CCN2/CTGF at the transcriptional level and also the processing of the same factor at the post-translational level, consistent with its definition as a moonlighting protein. However, the level of CD9 was higher in MMP3-KO-derived EVs compared to LuM1-EVs. The total protein concentration in the EV fraction was, not altered by MMP3 KO (Figure S5).

These data indicate that MMP3 positively regulates *CCN2/CTGF* transcription and the release of the C-terminal TSP1-CT fragment of CCN2/CTGF from the metastatic cancer cells, suggesting moonlighting regulatory roles for MMP3 at both transcriptional and post-translational levels.

### 2.6. EV-Mediated Intranuclear Transfer of MMP3 

**Figure 6:**
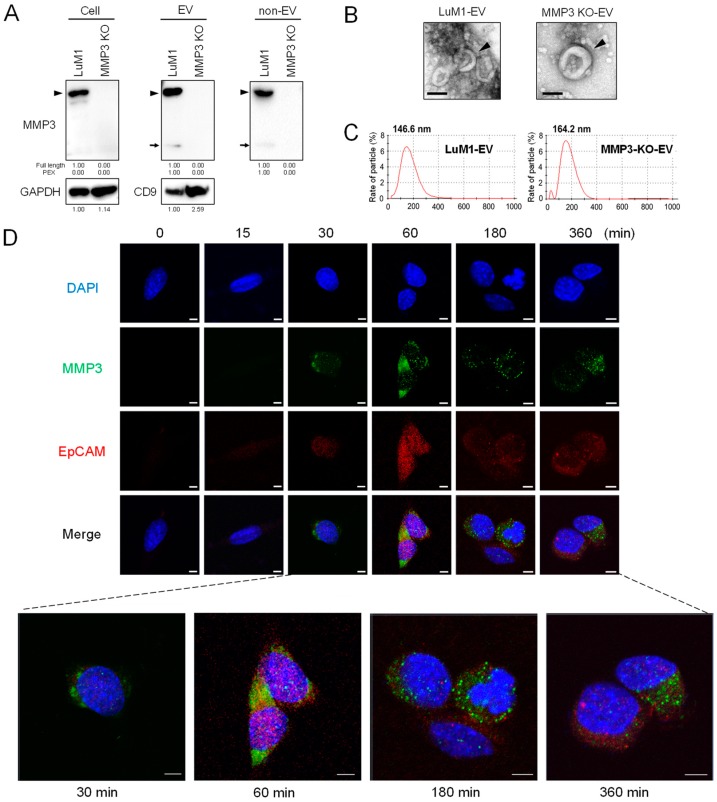
Oncosomal delivery of MMP3 to recipient cells intranuclearly. (**A**) Western blotting of MMP3 and CD9 in cell lysate, EVs and non-EV fractions. Arrowheads indicate full-length MMP3 (54 kDa). Arrows indicate the C-terminal PEX fragment of MMP3. The data of cell lysates were also shown in Figure 3F. (**B**) Representative TEM images of EV fractions derived from LuM1 and MMP3-KO cells. Arrowheads indicate vesicles with cup-shaped morphology. Scale bars, 100 nm. (**C**) Particle diameter distribution of EV fractions. (**D**) Oncosome-mediated intranuclear transfer of MMP3. LuM1-derived, MMP3-rich EVs were added into culture media of MMP3-KO recipient cells at a final concentration of 20 µg/mL. Cells were fixed at the indicated time points. Immunocytochemistry of MMP3 (green) and CD326/EpCAM (red) was carried out. Blue, DAPI. The images were taken under CLSM. Scale bars, 5 µm. The experiments were repeated twice in Figure 6A,D.

It has been shown that MMP3 possesses six NLS and extracellular MMP3 was able to translocate into the cellular nuclei where MMP3 positively regulated the *CCN2/CTGF* gene [28]. However, it was uncertain whether EVs were involved in the intranuclear transfer of MMP3. We therefore examined whether MMP3 enriched in LuM1-derived EVs was transferred into MMP3-null recipient cells and was located intranuclearly. This experimental system excluded the endogenous induction of MMP3 because the *Mmp3* gene was knocked out at the genome level in MMP3-KO recipient cells. We confirmed that MMP3 was undetectable in the cell lysate, EVs and non-EV fractions of MMP3-KO cells while being markedly detectable in those of LuM1 cells (Figure 6A; Figure S6).

Both LuM1 and MMP3-KO cells released EVs with cup-shaped morphology sized between 50 to 300 nm observed under TEM (Figure 6B). However, particle diameter distribution analysis revealed that the size of LuM1-EVs peaked at 146.6 nm, while that of MMP3-KO-EVs was enlarged to 164.2-nm diameter (Figure 6C), suggesting that the deletion of MMP3 might result in the retention of proteins on or in the vesicles.

Next, we investigated whether MMP3 in/on LuM1-EVs was molecularly transferred into MMP3-null recipient cells and nuclei. MMP3 was detected mainly in the cytoplasm but also in the nuclei of recipient cells from 30 min to 6 hours after the addition of LuM1-EVs (Figure 6D, green), suggesting that oncosomal MMP3 could be first taken into endosomes, and then some endosomally located molecules could escape to the nuclei.

It has been shown that a single-pass transmembrane glycoprotein CD326/EpCAM was often enriched in EVs derived from cancer stem cells (CSC, aka cancer-initiating cells (CIC)) and a cleaved intracellular domain of CD326/EpCAM was able to translocate intranuclearly to then exert transcriptional control [39,40]. Moreover, CD326/EpCAM is a category-1 EV marker protein defined in the *minimal information for studies of extracellular vesicles 2018* (MISEV2018) [37]. We therefore next examined the subcellular distribution of CD326/EpCAM after the addition of LuM1-EVs. CD326/EpCAM was barely detectable in MMP3-null cells at 0 min but was increased at 30 min to 6 hours after the addition of LuM1-EVs (Figure 6D, red). The co-localization of CD326/EpCAM and MMP3 was detected in the cytoplasm (supposed to be endosomes), but not in nuclei (Figure 6D, arrowheads). 

These data indicate that the pro-tumorigenic moonlighting metalloproteinase was deliverable from oncosomes to recipient cells and was able to accumulate in nuclei.

### 2.7. Oncosomal Transfer of MMP3 Trans-Activates and Induces CCN2/CTGF

**Figure 7:**
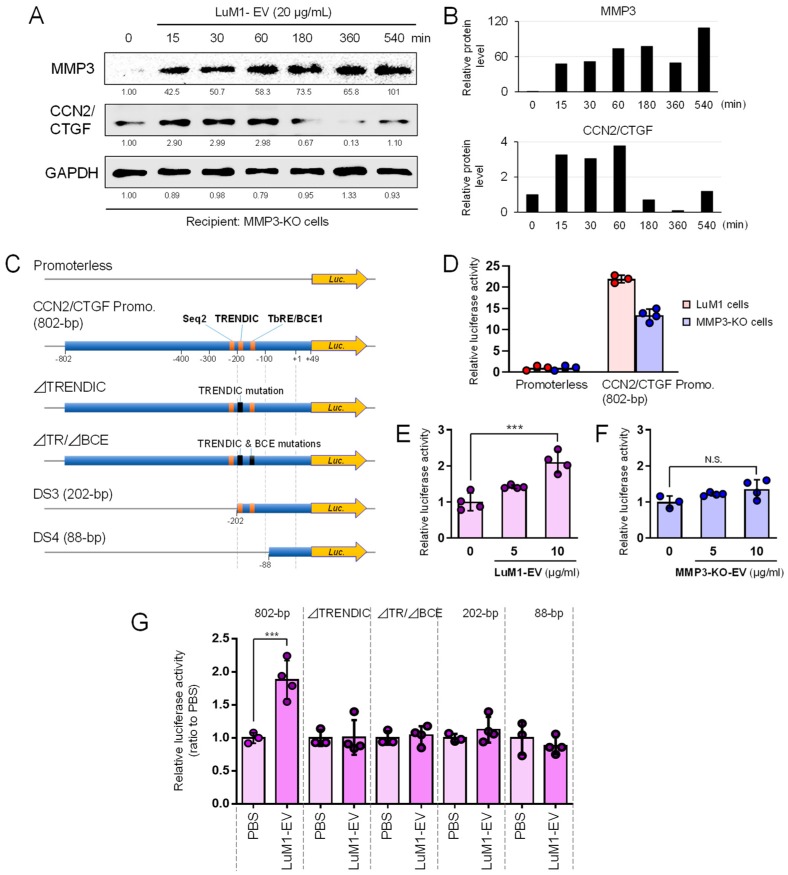
Oncosomal delivery of MMP3 induces CCN2/CTGF through transcriptional activation. (**A**,**B**) LuM1-derived MMP3-rich EVs were added to culture media of the MMP3-null recipient cells at a final concentration of 20 µg/mL. Cells were lysed at each time point (0, 15, 30, 60, 180, 540 min) after the addition of EVs. (**A**) Western blotting showing MMP3 and CCN2 in the recipient MMP3-KO cells. (**B**) The band intensities of MMP3 and CCN2, measured from panel A. The values relative to GAPDH are shown. (**C**–**G**) *CCN2/CTGF* promoter response to LuM1- or MMP3-KO-EVs. (**C**) Schemes of reporter constructs. Top to bottom; a promoterless construct, human 802-bp *CCN2/CTGF* promoter-driven luciferase (Luc.) reporter construct, ⊿TRENDIC mutant, ⊿TRENDIC/⊿BCE double mutant, and the 202-bp and 88-bp short promoter constructs (DS3 and DS4), respectively. (**D**) Relative activities of *CCN2/CTGF* promoter in LuM1 vs. MMP3-KO cells. phRL-TK was used as a control reporter vector. *** *p* < 0.001, n = 3 to 4. (**E**,**F**) Effects of LuM1- and MMP3-KO-EVs on *CCN2/CTGF* promoter activity in MMP3-KO cells. (**E**) LuM1-EVs or (**F**) MMP3-KO-EVs at final concentrations of 5 or 10 µg/mL or PBS was added to the serum-free culture media of MMP3-KO cells. *** *p* < 0.0001, n = 3 to 4. N.S., not significant. (**G**) Oncosomal responsivities of 802-bp CCN2/CTGF promoter and mutants. LuM1-EVs (10 µg/mL) or PBS was added to the serum-free culture media of MMP3-KO cells. *** *p* < 0.0001, n = 3 to 4. The experiments were repeated twice in Figure 7A,D,E.

To demonstrate oncosomal MMP3-driven induction of CCN2/CTGF, we examined whether the levels of MMP3 and CCN2/CTGF were altered by the transmission of LuM1-oncosomes into MMP3-null recipient cells. MMP3 was becoming stably detectable in the recipient cells from 15 min to 9 hours after EV addition (Figure 7A,B). The protein level of CCN2/CTGF (approximately 38 kD, potentially de novo synthesized full-length CCN2/CTGF) was also increased in recipient cells on 15 min to 1 hour after the addition of LuM1-EVs, although it decreased from 3 to 9 hours afterward (Figure 7A,B; Figure S7). The anti-CCN2/CTGF antibody detected an approximately 38-kD, potentially de novo, synthesized, full-length CCN2/CTGF in recipient cells, although LuM1-EVs did not contain the full-length CCN2/CTGF shown in Figure 5E. Consistently, CCN2/CTGF was under the detection limit in the EVs in LC-MS/MS analysis (Table 3).

To investigate the MMP3–CCN2 regulatory axis, we next examined whether the activity of the *CCN2/CTGF* promoter, containing MMP3-binding sequences, was altered by the deletion of the *Mmp3* gene or by the delivery of MMP3-rich or –null EVs. The human 802-bp *CCN2/CTGF* promoter-driven luciferase reporter activity was significantly lower in MMP3-KO cells than that in LuM1 cells (Figure 7C,D). MMP3-rich, LuM1-derived EVs (at a final concentration of 10 µg/mL) significantly increased the activity of the *CCN2/CTGF* promoter while MMP3-null EVs did not (Figure 7E,F). It was thus conceivable that MMP3 was essential for the oncosomal activation of the *CCN2/CTGF* promoter.

We previously reported that MMP3 was bound to the TRENDIC enhancer element [28,33] while also potentially bound to a basal control element (BCE, aka. the TGFβ response element (TbRE)) and to a sequence-2 (Seq-2) in the human *CCN2/CTGF* promoter [33,34]. We therefore examined whether the loss-of-function (LoF) mutation or deletion of these enhancer elements abolished the oncosomal responsivity of the *CCN2/CTGF* promoter. The LoF mutation in TRENDIC abolished the oncosomal response (Figure 7C,G). The shortening of the CCN2/CTGF promoter to 202-bp or 88-bp also abolished the oncosomal responsivity (Figure 7G). These data suggested that TRENDIC, as well as the upstream sequence (-802 to -202, including the Seq-2), were essential for the oncosomal responsivity of the *CCN2/CTGF* gene. Thus, MMP3 is deliverable from oncosomes into recipient cell nuclei, activates *CCN2/CTGF* gene transcription, and then induces CCN2/CTGF production.

### 2.8. Clinical Significance of MMP3 and CCN2/CTGF Expression

**Figure 8:**
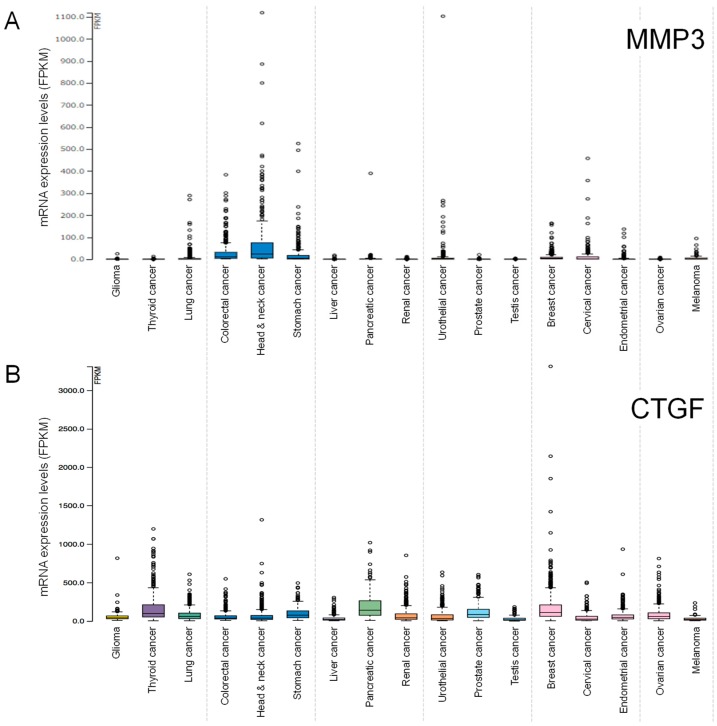
Expression levels of (**A**) *MMP3* and (**B**) *CCN2/CTGF* mRNA in patient-derived tumor specimens. Values indicate fragments per kilobase per million mapped reads (FPKM) of RNA-seq. Data were obtained from TCGA and Human Protein Atlas databases.

To investigate the clinical significance of *MMP3* and *CCN2/CTGF* gene expression, we searched the TCGA database of patient-derived tumor samples. *MMP3* mRNA expression was higher in head and neck cancer, stomach cancer, cervical cancer, pancreatic cancer, colorectal cancer, lung cancer, and urothelial cancer, compared to the ovarian, testis and other cancer types (Figure 8A).

*CCN2/CTGF* mRNA expression was higher in breast cancer, head and neck cancer, thyroid cancer, pancreatic cancer, glioma, endometrial cancer, ovarian cancer, renal cancer, compared to testis cancer and other cancer types (Figure 8B). 

**Figure 9:**
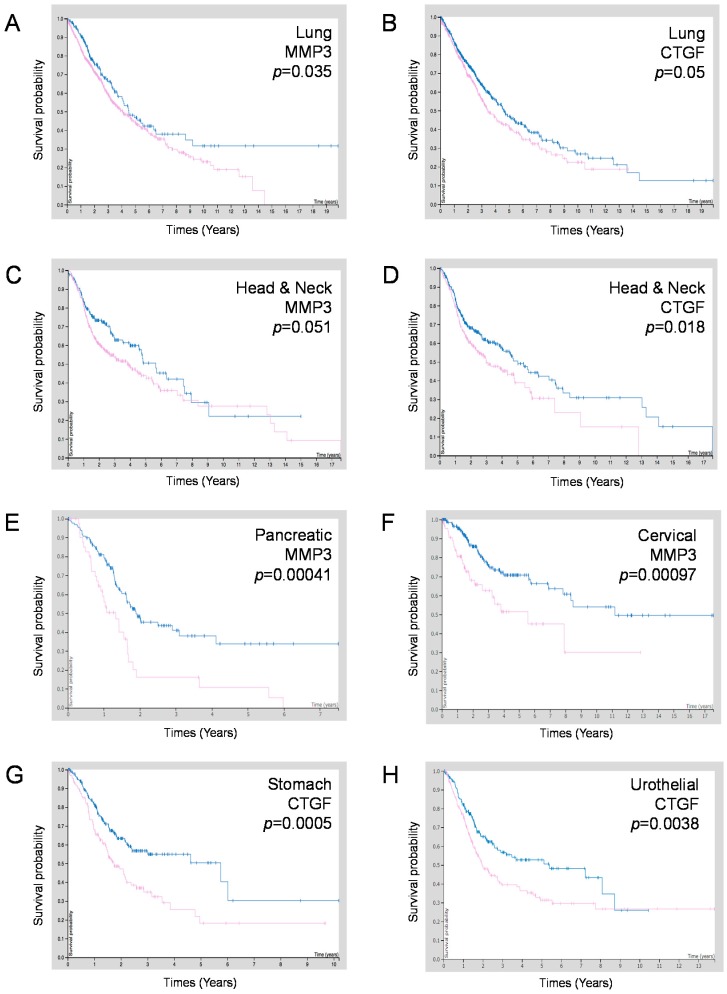
Kaplan–Meier survival curves of patient groups with MMP3 or CCN2/CTGF high-expression (purple line) vs. low-expression (blue line). (**A**,**B**) Head and neck cancer cases. (**C**,**D**) lung cancer cases. (**E**,**F**) Comparison between MMP3-high vs. -low expression groups in (**E**) pancreatic cancer cases and (**F**) cervical cancer cases. (**G**,**H**) Comparison between CCN2/CTGF-high vs. -low expression groups in (**G**) stomach cancer cases and (**H**) urothelial cancer cases. Data were obtained from TCGA and Human Protein Atlas databases.

To examine whether *MMP3* and/or *CCN2/CTGF* expression are correlated with the prognosis of patients suffering from these types of cancers, we carried out survival analyses using the Kaplan–Meier plot. High expression of *MMP3* and *CCN2/CTGF* were prognostically unfavorable in lung cancer and head and neck cancer (Table 4, Figure 9A–D). Moreover, a high expression of *MMP3* was prognostically unfavorable in pancreatic cancer (*p* = 0.00041), cervical cancer (*p* = 0.00097), and prostate cancer (*p* = 0.033) (Figure 9E,F; Table 3) while a high expression of *CCN2/CTGF* was prognostically unfavorable in stomach cancer (*p* = 0.0005), urothelial cancer (*p* = 0.0038), and colorectal cancer (*p* = 0.044) (Figure 9G,H; Table 4). 

The 5-year survival of MMP3-high pancreatic cancer patients was 11%, while that of the MMP3-low expression group was 34% (Table 5). The 5-year survival of MMP3-high cervical cancer patients was 51%, while that of the MMP3-low expression group was 71%. The 5-year survival of CCN2/CTGF-high stomach cancer patients was 18%, while that of the CCN2/CTGF-low expression group was 50% (Table 6). The 5-year survival of CCN2/CTGF-high urothelial cancer patients was 31%, while that of the MMP3-low expression group was 51%. 

These data indicate that mRNA expression levels of *MMP3* and *CTGF/CCN2* are useful as prognostic biomarkers in particular types of cancer.

## 3. Discussion

Our study indicates for the first time that aggressive cancer cells can release EVs enriched in MMP3 that potentially penetrate the tumor microenvironment, cross biological barriers, and become enriched in distant organs. Oncosomal MMP3 also plays transcriptional roles in the induction of the cell communication network factor CCN2/CTGF, a finding which may be important for understanding a mechanism of tumor–stroma progression and metastasis. Our study indicates that the rapidly metastatic tumor-derived, MMP3-rich EVs were rapidly transmissive, invasive to tissues and cells, and powerfully pro-tumorigenic, suggesting that MMP-rich EVs could be conceptual oncosomes involving primary and secondary tumorigenesis. The MMP3-rich EVs are coined as “oncosomes” remaining undefined as in the previous report [16]. In our current data, LuM1-EVs significantly promoted distant tumor growth through body fluids and caused cachexia in mice, accompanied by the systemic distribution of LuM1-EVs into multiple organs, including the liver, lung, and head. It was suggested that the injected EVs could directly target allografted tumor lesions. However, further experiments will be required to demonstrate how the injected EVs directly reach tumors by labeling and tracking EVs. In our in vitro study, LuM1-EVs delivering MMP3 altered the character of the recipient cells, represented by the immediate induction of an additional protumorigenic factor, CCN2/CTGF. Interestingly, the transferred MMP3 was sustained from 15 min to 9 h post-EV-addition period, whereas the induction of CCN2/CTGF was transient from 15 min to 1 hour, suggesting that CCN2/CTGF might be secreted from the cells upon synthesis or switched off by regulatory mechanisms. The current data showing the intranuclear delivery of MMP3 were confirmed under CLSM and consistent with our previous study which suggested that MMP3 possesses functional NLS and was able to translocate into the cellular nuclei, where the relocated MMP3 could positively regulate *CCN2/CTGF* and *HSP* genes [28,29,41]. Our current data indicate that LuM1-oncosomes can deliver pro-tumorigenic factor MMP3 from aggressive cancer cells to recipient cells, which MMP3 may reprogram. Indeed, the oncosomal MMP3 induced expression of CCN2/CTGF, consistent with a previous study demonstrating an MMP3-responsive element, TRENDIC, in the promoter region of the *CCN2/CTGF* gene [32,33,34]. Thus, tumor-derived oncosomes could have the potential to reprogram recipient cells by acting on the genome.

We have, in the present study, focused on the roles of EVs in the MMP3-CCN2 regulatory axis. We showed that the deletion of MMP3 resulted in the loss of the *CTGF* promoter activity. MMP3-rich LuM1-EVs rescued the *CTGF* promoter activity while MMP3-KO-EVs did not. Besides, we have shown that not only MMP3 but also MMP9 were crucial in LuM1 tumor progression [23]. MMP9 expression is positively regulated by oncogenic transcription factors such as STAT3, NF-kB, and β-catenin [42,43]. These factors may induce both MMP3 and MMP9 while being targetable by a single agent, Benztropine [42].

Our data also indicated that the MMP3-CCN2 regulatory axis could involve tumor–stroma interaction and progression. We have shown MMP3 to be localized in the border area and both sides of the tumor and stroma boundary in the LuM1 allograft model [23]. The abundant release of MMP3-EVs from LuM1, shown in the current study, is consistent with such molecular localization in vivo involving tumor–stroma interaction. However, there remains a lack of knowledge of CCN2/CTGF role/functions in cancer progression in vivo in the MMP3 switching on *CCN2/CTGF* gene as well as the role of the C-terminus of CCN2/CTGF cleaved by MMP3 and their spatial distribution during activation and tumor progression. Relevantly, MMPs were previously identified in EVs released from various cell types, some of which possessed proteolytic activities [44,45]. Relevantly, EVs derived from 8701-BC breast cancer cells and HT-1080 fibrosarcoma cells were reported to contain MMP9 in both pro- and mature forms with proteolytic activity [45,46]. Besides, MMP3 has been identified in/on EVs released from stromal cells, such as mesenchymal stromal cells (MSC) and adipocytes [47,48]. Moreover, it has been shown that cancer exosomes could trigger MSC differentiation into pro-angiogenic and pro-invasive myofibroblasts, secreting high levels of matrix-regulating factors (MMP-1, -3, and -13), VEGF-A, and HGF [49]. A recent study reported MMP3 to be up-regulated in both stromal fibroblasts and cancer cells by oxidative stress [50]. Moreover, CCN2/CTGF can be produced from both tumor and stromal cells. MSC-derived CCN2/CTGF promoted tongue squamous cell carcinoma progression [51]. Another study reported that tumor-derived CCN2/CTGF could mediate tumor–stromal interactions which accelerate hepatocellular carcinoma progression [52]. Notably, it has recently been postulated that CCN2/CTGF might contribute to colorectal cancer progression, especially in a fibrotic consensus molecular subtype [53]. Thus, MMP3, CCN2/CTGF, and their EVs can be produced on both sides of the interaction to promote tumor–stroma malignant conversion. It is conceivable that the knockout of MMP3 can efficiently interrupt the pro-malignant communication between tumor and stroma.

We also observed that the levels of CD326/EpCAM, a marker of CSCs and category-1 marker protein of EVs, increased in the cells and nuclei upon oncosomal transfer, although it is uncertain whether CD326/EpCAM was transferred from EVs or de novo synthesized in recipient cells. Nevertheless, CD326/EpCAM was detected in the cytoplasm and nuclei from 30 min to 6 hours post-EV-addition period, suggesting that MMP3 and CD326/EpCAM were co-transferred from the LuM1-EVs to recipient cells. It has been shown that CD326/EpCAM is a single-pass transmembrane glycoprotein, often enriched in EVs derived from CSC/CIC, while the CD326/EpCAM intracellular domain can translocate to the nucleus forming a transcriptional complex with β-catenin, a process which activates the stemness genes [39,40]. Thus, the LuM1-derived oncosomes could contain MMP3 and CD326/EpCAM, whose transfer may induce stemness in recipient cells in the local tumor microenvironment and distant organs. There are two potential mechanisms of MMP3 transmission, including (i) six basic amino acid clusters as functional NLS in MMP3 [28,29], which could work as endosomal membrane-permeable, membrane-disrupting translocase/flippases as in the Trans-Activator of Transcription Protein (TAT) peptide, also called the Cell-Penetrating Peptide (CPP) [54]; and/or (ii) proteolysis-dependent endosomal escape, a mechanism seen in other proteinases such as cathepsins that are essential for the endosomal escape of viruses [55,56]. Thus, MMP3 protease activity may promote EV transmission and invasion into cells by degrading ECM, cell surface receptors, and cytoskeletal proteins on/in recipient cells. 

Our data also touch upon key proteolytic activities of the metalloproteinase on/in the LuM1 cells and their secretome. Fragments of MMP3 as a marker of MMP proteolytic activity were found in LuM1 cellular, EVs and non-EV fractions in significant amounts. It has been shown that metalloproteinases including MMP3 possess a self-cleavage, self-activation mechanism [23,28]. Therefore, it is conceivable that the PEX fragment in EVs is a functional remnant (metabolite) indicating on-EV proteolysis by metalloproteinases. The C-terminal half fragments of CCN2/CTGF on EVs and in non-vesicular extracellular fractions are also potentially functional remnants generated by the metalloproteinase activity. Consistently, it has been shown that active MMPs cleaved CCN2/CTGF to generate an N-terminal half fragment (IGFBP-VWC modules) and a C-terminal half fragment (TSP1-CT modules), a bioactive fragment essential for angiogenesis [27,30,57,58]. Moreover, the C-terminal half fragment of CCN2/CTGF promoted osteoclastogenesis [31]. Thus, the EV-associated CCN2/CTGF fragments, found in our study, may play a key functional role in tumor–stroma interaction. However, our study has not determined pro-tumorigenic roles and binding partners for CCN2/CTGF. CCN2/CTGF can bind with many partners including CCNs homo-/hetero-dimerization, integrins, EGFR, Notch, LRP1/6, fibronectin, BMP2/4, TGFβ, FGF2, or aggrecan [59]. Moreover, MMPs cleave many extracellular proteins including ECM (collagen, proteoglycan, etc.), matricellular proteins (osteopontin [60], CCN2/CTGF [30]), transmembrane proteins (E-cadherin, pro-HB-EGF), growth factors (latent TGFβ), and an immune checkpoint protein (PD-L1) [61]. Thus, MMP3 and CCN2/CTGF may regulate matricellular balance in the vesicular microenvironment by its moonlighting properties. 

Our data also indicate the fragmentation of MMP3 itself to generate the PEX fragment. MMPs have a self-cleavage mechanism, by which active MMP3 might cleave themselves on EVs. Indeed, active MMP3 (50-kD band) was found just below the full-length MMP3 in both cell lysate and EV fractions in Figure 1C and the PEX fragment could be generated by additional cleavage. Moreover, different MMP family proteins can cleave each other. We have shown that MMP9 was also highly expressed in and released from LuM1 compared to Colon26 [23,43]. Therefore, MMP9 may potentially cleave MMP3 to generate the PEX. Notably, these MMPs have been often found in soluble fractions as well, and thus can approach and cleave EV surface proteins.

Our data also touch upon EV cargo properties which are different among Colon26, LuM1, and MMP3-KO-LuM1 cells. We previously showed that EVs were more abundantly released by LuM1 than Colon26 cells [35], suggesting that LuM1 might release oncosomes more efficiently than Colon26. CD9 is a member of the tetraspanin family, highly palmitoylated, and essential for intercellular adhesion by forming a complex with CD326, integrins, IgM, and cholesterol [62,63,64,65,66]. In the present study, for the analysis of CD9 levels in EV fractions from Colon26 vs. LuM1, we applied protein samples from the same number of cells (1.2 × 10^5^ cells) and found that the LuM1-EVs contained a high proportion of CD9 in EVs compared to Colon26-EVs. EVs often contain HSPs, category-2a marker of EVs [37] as well as a marker of stressome [11]. Interestingly, Colon26-EVs contained HSP90α at higher levels than LuM1-EVs, in contrast to CD9 (data not shown) [38]. Therefore, we are currently investigating the balance between oncosomes and HSP containing stressomes. Moreover, the deletion of MMP3 tended to increase the size of EVs (Figure 6) and the volume of EV proteins (Figure S5), suggesting that proteolytically active MMPs cleave out proteins on the vesicles in the extracellular space while the deletion of MMP3 resulted in the retention of proteins on such vesicles. Therefore, it is conceivable that LuM1-EVs contained substrate proteins that are cleavable by MMP3 on the vesicles. Thus, the presence or absence of MMPs on the vesicles markedly alters the properties of the EVs at the proteome and functional levels.

## 4. Materials and methods

### 4.1. Cells

A murine colon cancer cell line Colon26 (aka CT26) [23,67] and a murine calvaria-derived osteoblastic cell line MC3T3-E1 [68] were obtained from Cell Bank at RIKEN BioResource Research Center (Tsukuba, Japan). A rapidly metastatic subline, LuM1, was generated from Colon26 as described previously [23,35,36,42,43]. MMP3-knockout LuM1 (MMP3-KO) cells were generated using the genome-editing method as described below. A murine macrophage-like cell line RAW-D (a subline of RAW264.7) was kindly provided by Prof. Toshio Kukita (Kyushu University, Fukuoka, Japan) [69]. Colon26, LuM1, MMP3-KO LuM1, and MC3T3-E1 were maintained in RPMI1640 with 10% fetal bovine serum (FBS) and penicillin, streptomycin, and amphotericin B. RAW-D cells were cultured in minimum essential medium (MEM; Wako Pure Chemicals, Osaka, Japan) containing 10% FBS, supplemented with penicillin (100 U/mL) and streptomycin (100 mg/mL).

### 4.2. Genome Editing

DNA oligonucleotides used for gRNA synthesis were designed with the GeneArt™ CRISPR gRNA Design Tool. Among many candidates, we chose gRNA #1 and #2 sequences (Table 1) targeting exon 1 and exon 2, respectively, of the *Mmp3* gene in murine chromosome 9 (position 7445958) without any mismatch, but with 2–3 nucleotide mismatch on other chromosomal sequences to minimize off-target. We finally used the gRNA #1 with a higher cleavage efficiency than gRNA #2.

The gRNAs were then synthesized using the GeneArt™ precision gRNA synthesis kit (ThermoFisher, Waltham, MA, USA). CRISPR RNA (crRNA)/trans-activating crRNA (tracrRNA) hybridization and RNP complex formation was done according to the manufacturer’s instructions. For transfection, 8 × 10^4^ cells were seeded in 24-well plates and incubated overnight. Cells were then transfected with the RNP composed of gRNA (125 ng) and recombinant Cas9 nuclease (rCas9; 500 ng) using the CRISPRmax transfection reagent (ThermoFisher). The genomic cleavage efficiency was measured by a PCR-based method using the GeneArt^®^ Genomic Cleavage Detection (GCD) kit (ThermoFisher) according to the manufacturer’s instructions. Cells were corrected at 48 h post-transfection and PCR was carried out. The amplicon was loaded to 2% agarose gel electrophoresis. The cleavage efficiencies were calculated based on the relative band intensities, and quantified using Image J. Isolation of the single clone was carried out by dilution cloning method using a 96-well plate. Genomic DNA was extracted from each clone by using a DNeasy Blood & Tissue Kit (Qiagen, Hilden, Germany). Exon 1 of *Mmp3* in each clone were amplified using Blend Taq Plus (Toyobo, Osaka, Japan) and GCD primer sets under the following conditions in the thermal cycler: an initial denature at 95 °C for 10 min, 30 cycles (at 95 °C for 30 s, at 55 °C for 30 s, and 72 °C for 30 s), followed by a final extension at 72 °C for 7 min. PCR products were cleaned up using ExoSAP-ITTM Express PCR Product Cleanup Kit (ThermoFisher). The sequences of each clone were analyzed by Sanger sequencing method. To establish a bi-allelic deletion, gRNA/rCas9 and pEGFP-c1 vector were cotransfected into a heterogenic deletion clone (-43; clone #14) in three different conditions shown in Figure S1c by using NEPA21 electroporator (NEPA Gene, Ichikawa, Japan). Cells were cultured and selected within G418 antibiotics at a concentration of 0.5 mg/mL for 20 days. DNA extraction, PCR, and sequencing were performed as described above.

### 4.3. Isolation of EV

EV fraction was prepared using a modified polymer-based precipitation method as described [14]. Briefly, cells growing in two 10-cm dishes were washed with PBS (-), and then further cultured in 4 mL of serum-free medium per dish for 2 days. Cell culture supernatant was centrifuged at 2000× *g* for 30 min at 4 °C to remove detached cells. The supernatant was then centrifuged at 10,000× *g* for 30 min at 4 °C to remove cell debris. In the PBP method, the supernatant (8 mL) was concentrated to less than 1 mL by using an Amicon Ultra-15 Centrifugal Filter Devices for M.W. 100k (Merk Millipore, Burlington, MA, USA). The concentrate was applied to Total EV fraction Isolation (ThermoFisher, Waltham, MA, USA). The pass-through was concentrated using an ultrafiltration device for molecular weight 10 kD and used as a non-EV fraction. The EV fraction was suspended in 100 µL PBS (-). Protein concentration was measured using a micro BCA protein assay kit (ThermoFisher). 

### 4.4. Transmission Electron Microscopy

As described previously [15], a 400-mesh copper grid coated with formvar/carbon films was hydrophilically treated. The EV suspension (5–10 µL) was placed on Parafilm, and the grid was floated on the EV liquid and left for 15 min. The sample was negatively stained with 2% uranyl acetate solution for 2 min. EV fraction on the grid was visualized with 20,000 times magnification with an H-7650 transmission electron microscope (Hitachi, Tokyo, Japan) at the Central Research Laboratory, Okayama University Medical School. 

### 4.5. Particle Diameter Distribution

As described previously [4], forty microliters of EV fraction within PBS (-) was used. Particle diameters of the EV fractions in a range between 0 and 1000 nano-diameters were analyzed in Zetasizer nano ZSP (Malvern Panalytical, Malvern, UK).

### 4.6. Immunocytochemistry and Confocal Laser Scanning Microscopy

Cells were seeded at concentrations of 4.0 × 10^4^ per well in a 24-well-plate and then incubated overnight. LuM1-derived EV fraction was added to culture media in a 24-well plate at a concentration of 20 μg/mL, and then cells were cultured for 0, 15, 30, 60, 180, 360, and 540 min. Cells were then fixed with 4% paraformaldehyde in PBS for 10 min at each time point after the addition of EVs. Immunocytochemistry and CLSM were carried out as described [23,28]. Briefly, cells were permeabilized with 0.5% Tween-20 in PBS for 5 min. The fixed cells were blocked in 10% normal goat serum solution for 30 min and then incubated overnight at 4 °C with rabbit anti-MMP3 antibody (EP1186Y, ab52915, Abcam) and mouse anti-CD326/EpCAM antibody (EGP40/1110; Abgent, San Diego, CA, USA) in 10% normal goat serum solution. Cells were then incubated with anti-rabbit IgG AlexaFluor488 [Cell Signaling Technology (CST), Danvers, MA] or anti-mouse IgG AlexaFluor594 (CST) for 1 hour at room temperature. Cellular Nuclei were stained with 4′, 6-diamidino-2-phenylindole (DAPI; Invitrogen, Carlsbad, CA, USA). The fluorescence was analyzed using a CLSM imaging system LSM 780 META (Carl Zeiss, AG, Jena, Germany). To define MMP3 or CD326/EpCAM positivity, the fluorescence intensity of the cells without the primary antibodies were subtracted as background.

### 4.7. EV-Mediated Molecular Transfer

The EV-mediated molecular transfer experiments were performed as previously described [6]. Colon26 cells were seeded at a concentration of 4.0 × 10^4^ cells per well in a 24-well plate and incubated overnight. LuM1-derived EV fraction (8 or 20 μg/mL) was added to the culture media. Cells were cultured for 9 h and the medium was replaced with serum-free medium. Cells were cultured for 2 days and then lysed in RIPA buffer for western blotting.

MMP3 KO cells were seeded at a concentration of 1.2 × 10^6^ cells per dish in a 10 cm dish and incubated overnight. LuM1-derived EV fraction was added to the culture media at a final concentration of 20 μg/mL. The recipient cells were lysed with RIPA buffer at 0, 15, 30, 60, 180, 360, and 540 min post-EV-addition period for Western blotting.

### 4.8. Luciferase Reporter Assay

Luciferase assay was carried out as previously described [28,32,34,70]. Briefly, LuM1 or MMP3 KO cells were seeded at 2 × 10^4^ cells per well in a 96-well plate and cultured for 24 h in RPMI 1640 medium with 10% FBS. The human *CCN2/CTGF* promoter-driven firefly luciferase reporter plasmid (pTS589) [32] and mutant plasmids including ⊿TRENDIC [33], ⊿TRENDIC/⊿BCE [28,33], pDS3 (202-bp promoter), and pDS4 (88-bp promoter) were described previously [32]. Any one of these reporter plasmids (100 ng/well) or the promoterless vector PGV-B2 (Nippon Gene, Tokyo, Japan) was cotransfected with a *Renilla* luciferase control vector phRL-TK (Promega, Madison, WI) at 20 ng/well using Lipofectamine 3000 transfection reagent (ThermoFisher). Media were replaced with RPMI 1640 without FBS and EVs were added to the media at concentrations of 0, 5 or 10 µg/mL and cells were incubated for 24 h. Cells were lysed and luciferase activities were measured using the Dual-Luciferase Reporter Assay System (Promega, Madison, WI, USA). 

### 4.9. EV Transmission Imaging

EV labeling and transmission experiments were carried out as described previously [4,35]. The EV fraction (20 μg) was incubated with 10 μM BODIPY TR Ceramide (ThermoFisher) for 20 min at 37 °C. Cells (Colon26, LuM1, RAW-D, or MC3T3-E1) were treated with the BODIPY-labeled EVs derived from Colon26 or LuM1 at 11.5 μg/mL for 24 h. Cells were fixed with 4% paraformaldehyde in PBS (-) for 10 min and stained with ActinGreen488 (ThremoFisher). Nuclei were counterstained using DAPI. Fluorescence images of random three fields (2430 μm^2^/field) were taken using a Floid^®^ Imaging Station (ThermoFisher) and fluorescence-positive cells in each field were counted using ImageJ.

### 4.10. In Vivo Imaging

In vivo imaging experiments were performed as previously described [71]. EV fraction (15 µg) was incubated with 5 µM Cy7 Mono NHS Ester (GE Healthcare, Buckinghamshire, UK) for 90 min at 37 °C. The unincorporated dye was removed using Exosome Spin Columns (MW. 3000) (ThermoFisher). Animal experiments were performed in compliance with the guidelines of the Ethics Committee of Animal Care and Experimentation of Tokushima University (approval number T29-31). Eight-week-old female BALB/c mice (CLEA, Tokyo, Japan) were administered with 15 µg Cy7-labeled EVs per mouse by intraperitoneal injection. At 30 min after the injection, Cy7 fluorescence in the various organs of mice was analyzed by the IVIS Spectrum imaging system (Caliper Life Sciences, A PerkinElmer Company, Hopkinton, MA, USA).

### 4.11. Tumor Allograft to Mice

Animal experiments were performed according to the guidelines for the care and use of laboratory animals approved by Okayama University (OKU-2018761) and the Japanese Pharmacological Society. Subcutaneous allograft was performed as previously described [6,23]. Colon26 cells (5.0 × 10^5^ cells in 0.5 mL PBS) were transplanted subcutaneously into a side abdominal wall of each 6- to 7-week-old female BALB/c mouse. Five micrograms per 0.5 mL (protein concentration) of the EV fraction derived from Colon26 or LuM1, or 0.5 mL PBS was injected i.p. from Day 3 to 13, every other day, six times. On Day 21 after transplantation, the mice were sacrificed and the weight of each subcutaneous tumor was measured.

### 4.12. Cell Proliferation

For the analysis of cell proliferation, cells were seeded at a concentration of 2.5 × 10^3^ cells/well in a 96-well plate. The number of cells at 1 to 5 days post-seeding period was counted using Countess automated cell counter (ThermoFisher). The medium was replaced with a fresh one every 3 days during the analysis of cell proliferation.

### 4.13. Migration/Invasion Assay

Migration and invasion assays were performed as previously described [23,43]. Uncoated and Matrigel-coated culture systems (Becton–Dickinson, Franklin Lakes, NJ) were used for in vitro migration and invasion assays, respectively. Cells were seeded at concentrations of 2.5 × 10^4^ per well in a 24-well-plate into the upper chambers of transwells. Cells that migrated or invaded through the pores to the lower surface of the filter were fixed, stained using Diff-Quick stain (Sysmex, Kobe, Japan), and counted after 24 hours of the migration period. 

### 4.14. Western Blot Analysis

Western blotting was performed as previously described [23,72]. Briefly, cells were lysed in a RIPA buffer (1% NP-40, 0.1% SDS, and 0.5% deoxycholate, and EDTA-free protease inhibitor cocktail in PBS) using 25-gauge syringes. The same protein amounts or the same number of cells were subjected to sodium dodecyl sulfate–polyacrylamide gel electrophoresis (SDS-PAGE), followed by transfer to a polyvinylidene fluoride (PVDF) membrane using wet- and semi-dry methods where appropriate. The membranes were blocked in 5% skim milk in Tris-buffered saline containing 0.05% Tween 20 for 60 min unless otherwise specified, and incubated with a rabbit monoclonal anti-MMP3 antibody (EP1186Y, ab52915; Abcam, Cambridge, UK) or a rabbit monoclonal anti-CD9 antibody (EPR2949, ab92726, Abcam). For CCN2/CTGF, blocking was performed in 10% skim milk overnight and a rabbit polyclonal anti-CCN2/CTGF antibody (ab6992, Abcam) was reacted for 2 days. The membranes were incubated with horseradish peroxidase (HRP)-conjugated secondary antibodies. For GAPDH, HRP-conjugated anti-GAPDH mouse monoclonal antibody (HRP-60004, Proteintech, Rosemont, IL, USA) was used. Blots were visualized with the ECL substrate.

### 4.15. Microarray Analysis and Bioinformatics

Microarray analysis was performed as described [23,41]. Raw data were submitted to the Gene Expression Omnibus (GEO) database repository; accession ID: GSE97166; Colon26, GSM2553008; LuM1, GSM2553009; NM11, GSM2553010. Gene expression was analyzed using MeV 4.0 software (http://www.Tm4.org/mev.html) for the generation of heatmaps. 

### 4.16. Real-Time qRT-PCR

Total RNA preparation and qRT-PCR was carried out as described previously [23,68]. The miRNeasy mini kit (Qiagen) was used with DNase (Qiagen). The total RNA concentration was measured by using a micro spectrometer K2800 (Beijing Kaiao, Beijing, China). cDNA synthesis was carried out by using iScript™ cDNA Synthesis Kit (Bio-Rad, Richmond, CA, USA). The primers are the blend of oligo (dT) and random primers. Specific primer pairs for *Mmp3, CCN2/CTGF*, and *Gapdh* (Table 7) were used for real-time PCR with an iQ SYBR Green PCR mixture (Bio-Rad). Relative mRNA levels to *Gapdh* mRNA levels were quantified by the ΔΔCt method using the formula–fold change = 2^−ΔΔCt^. PCR reaction was carried out in triplicate and mean values were calculated with the mean ± S.D. of the biological triplicates presented.

### 4.17. Patient-Derived Tumor Samples

The co-expression correlation dataset of colorectal adenocarcinoma with 632 patient-derived 632 samples from TCGA was analyzed using cBioPortal.

The mRNA expression levels and Kaplan–Meier survival curves of patient groups with MMP3 or CCN2/CTGF high- vs. low-expression in various types of cancers were analyzed within the data from Human Protein Atlas and TCGA databases.

### 4.18. Statistical Analysis

Statistical significance was calculated using GraphPad Prism and Microsoft Excel. Three or more mean values were compared using one-way analysis of variance (ANOVA), while comparisons of two were done with an unpaired Student’s *t*-test. *p* < 0.05 was considered to indicate statistical significance. Data were expressed as Mean ± SD unless otherwise specified.

## 5. Conclusions

In conclusion, EVs enriched with moonlighting metalloproteinase MMP3 are transmissive, pro-tumorigenic, and induce cellular communication network factor 2, which involves tumor–stromal interaction and progression. In the present study, oncosomes were defined by key characters such as the MMP-rich, pro-tumorigenic, and highly transmissive properties of tumor-derived EVs.

## Figures and Tables

**Table 1:** The sequence of gRNA.

Name of Primers	5′ to 3′ Sequences
gRNA #1	TCCCTTGCACTGTCATGCAA
gRNA #2	CAAGGCCATAGTAGTTTTCT

**Table 2:** Genome editing efficiencies.

Checkpoints	First Edit	Second Edit
Cells	LuM1	Clone #14
Method of Cas9/gRNA transfection	Reagent	Electroporation
The number of clones sequenced	21	8
Mono-allelic INDEL	3	0
Bi-allelic INDEL	0	6
Negative (not edited)	18	2
Changes in the number of nucleotides	+16, −43, −19	−1
Genome editing efficiency	14.3%	75%

**Table 3:** Protein MS/MS scores of EV fractions derived from LuM1 vs MMP3-KO cells.

Protein Name	LuM1-Derived EVs	MMP3-KO Cell-Derived EVs
MMP3/stromelysin 1 (STR1)	23.66	N.D.
CCN2/CTGF/FISP12	N.D.	N.D.
ACTBL2	20.21	19.79

N.D. not detected.

**Table 4:** *MMP3* and/or *CCN2/CTGF* expression correlated with the prognosis of cancer patients.

Tumor Region	MMP3	CCN2/CTGF	Correlation
Lung	*p* = 0.035 *	*p* = 0.05 *	MMP3-high, CTGF-high; poor prognosis
Head & Neck	*p* = 0.051	*p* = 0.018 *	MMP3-high, CTGF-high; poor prognosis
Melanoma	*p* = 0.071	*p* = 0.17	MMP3-high, CTGF-high; poor prognosis
Glioma	*p* = 0.35	*p* = 0.13	MMP3-high, CTGF-high; poor prognosis
Pancreatic	*p* = 0.00041 ****	*p* = 0.24	MMP3-high; poor prognosis
Cervical	*p* = 0.00097 ****	*p* = 0.14	MMP3-high; poor prognosis
Prostate	*p* = 0.033 *	*p* = 0.21	MMP3-high; poor prognosis
Stomach	*p* = 0.32	*p* = 0.0005 ****	CTGF-high; poor prognosis
Urothelial	*p* = 0.23	*p* = 0.0038 ***	CTGF-high; poor prognosis
Colorectal	*p* = 0.0016 ***	*p* = 0.044 *	CTGF-high; poor prognosis
Breast	*p* = 0.23	*p* = 0.29	CTGF-high; poor prognosis
Endometrial	*p* = 0.0051 **	*p* = 0.057	MMP3-high, CTGF-high; better prognosis

The data were expressed as a log-rank test *p*-value. * *p* < 0.05, ** *p* < 0.01, *** *p* < 0.005, **** *p* < 0.001. Expression levels in each case were shown in Figure 8. Kaplan–Meier plots are shown in Figure 9.

**Table 5:** Five-year survival in MMP3-high vs. -low expression groups.

Site	MMP3 High	MMP3 Low
**Pancreatic cancer**	11%	34%
**Cervical cancer**	51%	71%

**Table 6:** Five-year survival in CTGF-high vs. -low expression groups.

Site	CTGF High	CTGF Low
**Stomach cancer**	18%	50%
**Urothelial cancer**	31%	53%

**Table 7:** The sequence of primers for RT-qPCR.

Name of Primers	5′ to 3′ Sequences
m Gapdh Fw	ACCACAGTCCATGCCATCAC
m Gapdh Rv	TCCACCACCCTGTTGCTGTA
m Mmp3 Fw	ACCAACCTATTCCTGGTTGCTGCT
m Mmp3 Rv	ATGGAAACGGGACAAGTCTGTGGA
m CCN2/CTGF Fw	CTCCACCCGAGTTACCAATGACAA
m CCN2/CTGF Rv	CCAGAAAGCTCAAACTTGACAGGC

## Supplementary Materials

The following are available online at https://www.mdpi.com/2072-6694/12/4/881/s1, Figure S1. Full images of Western blotting of MMP3 (A), CD9 (B), and GAPDH (C), supporting Figure 1C; Figure S2. Western blotting of MMP3 and GAPDH in recipient Colon26 cells after the addition of LuM1-EVs; Figure S3. Basic data of genome editing, supporting Figure 3; Figure S4. Full images of Western blotting of CCN2/CTGF (A), CD9 (B), and GAPDH (C), supporting Figure 4E; Figure S5. Protein concentrations in each EV fraction derived from Colon26, LuM1, and MMP3-KO cells; Figure S6. Full images of Western blotting, supporting Figure 3F and Figure 6A; Figure S7. Full images of Western blotting of MMP3 (A), CCN2/CTGF (B), and GAPDH (C), supporting Figure 7A.

## Abbreviations

CCN2	cellular communication network factor / CCN family 2
CIC	cancer-initiating cells
CLSM	confocal laser scanning microscopy
CSC	cancer stem cells
CTGF	Connective tissue growth factor
CRISPR	clustered regularly interspaced short palindromic repeat
ECM	extracellular matrix
EV	Extracellular vesicle
IVIS	in vivo imaging system
MMP	Moonlighting metalloproteinase
MV	Microvesicle
PEX	hemopexin-like repeat
