# Supplementary material for: Extracellular Vesicles Enriched with Moonlighting Metalloproteinase Are Highly Transmissive, Pro-Tumorigenic, and Trans-Activates Cellular Communication Network Factor (CCN2/CTGF): CRISPR against Cancer
Source: Cancers (Basel). 2020 Apr 4;12(4):881. doi: 10.3390/cancers12040881 (PMC7226423; doi:10.3390/cancers12040881)
Supplement: Supplementary file 1 [file cancers-12-00881-s001.pdf]

Supplementary information

Extracellular Vesicles enriched with Moonlighting Metalloproteinase are highly Transmissive, Pro-Tumorigenic, and *Trans*-Activates Cellular Communication Network Factor (*CCN2/CTGF*): CRISPR against Cancer

Yuka Okusha, Takanori Eguchi, Manh T. Tran, Chiharu Sogawa, Kaya Yoshida, Mami Itagaki, Eman A. Taha, Kisho Ono, Eriko Aoyama, Hirohiko Okamura, Ken-ichi Kozaki, Stuart K. Calderwood, Masaharu Takigawa, and Kuniaki Okamoto

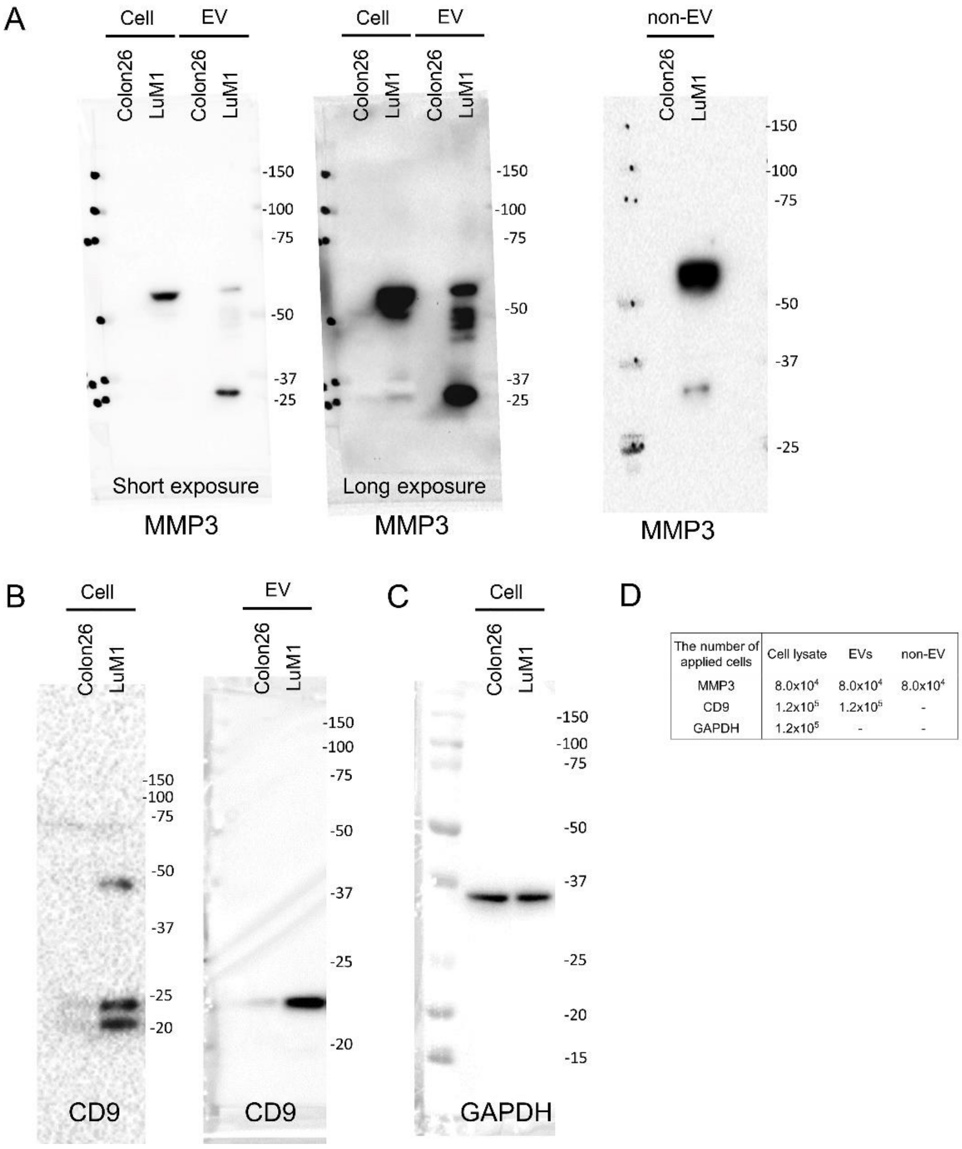

D

The number of applied cells	Cell lysate	EVs	non-EV
MMP3	8.0×10^4	8.0×10^4	8.0×10^4
CD9	1.2×10^5	1.2×10^5	-
GAPDH	1.2×10^5	-	-

Figure S1. Full images of Western blotting of MMP3 (A), CD9 (B), and GAPDH (C), supporting Figure 1D. (D) The number of cells applied to each lane was shown.

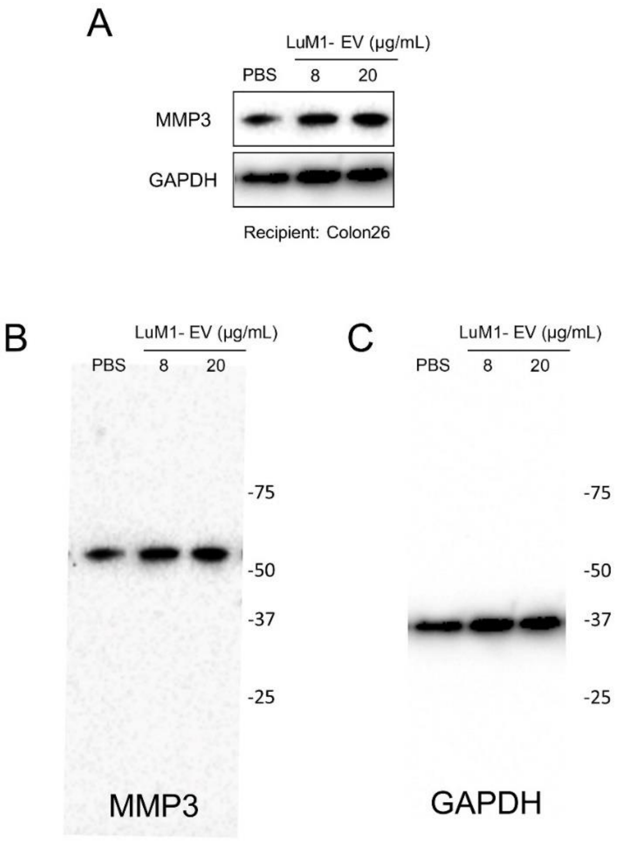

Figure S2. (A) Western blot showing MMP3 and GAPDH in recipient Colon26 cells after the addition of LuM1-EVs. LuM1-EVs (8 or 20 μg/mL) enriched with MMP3 were added to culture media of the recipient Colon26 cells (MMP3 low) for 9 h. (B, C) Full images of western blotting of MMP3 (B) and GAPDH (C). To each well, 50 μg was applied.

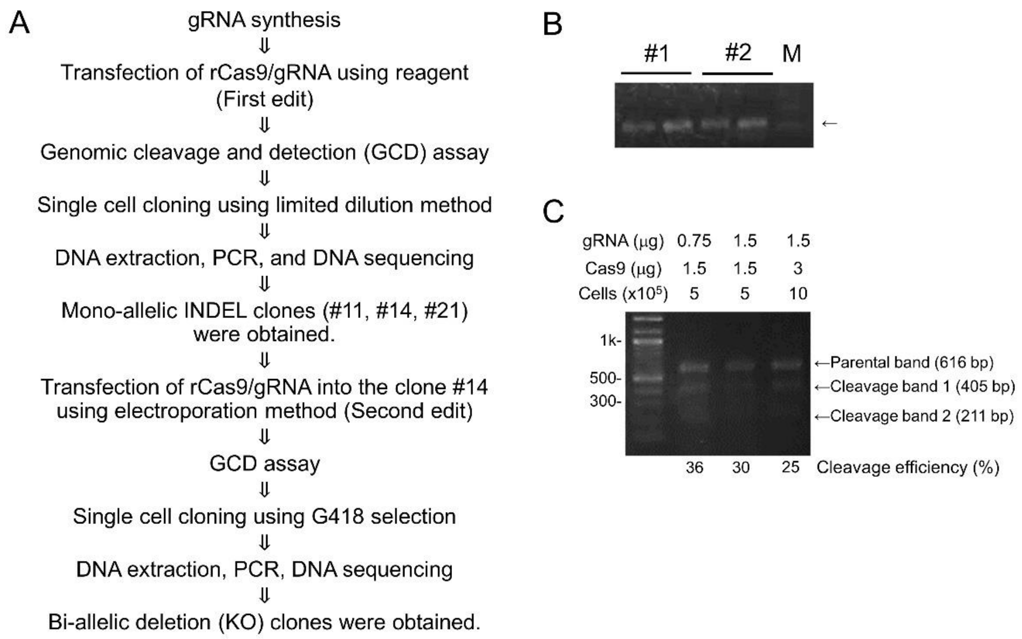

Figure S3. Basic data of genome editing, supporting Figure 3. (A) A flow chart of genome editing. (B) Agarose gel electrophoresis of the synthesized gRNA. #1, gRNA that targets exon 1 in mouse *Mmp3* gene, used in the present study. #2, gRNA that targets exon 2 in mouse *Mmp3* gene, not used in the present study. M, molecular weight marker. (C) Genome cleavage and detection (GCD) assay. The transfection conditions including the amount of gRNA, Cas9, and cells were shown on the top. Genome cleavage efficiencies were shown on the bottom. Parental band, cleavage band 1 and 2 were indicated by arrows.

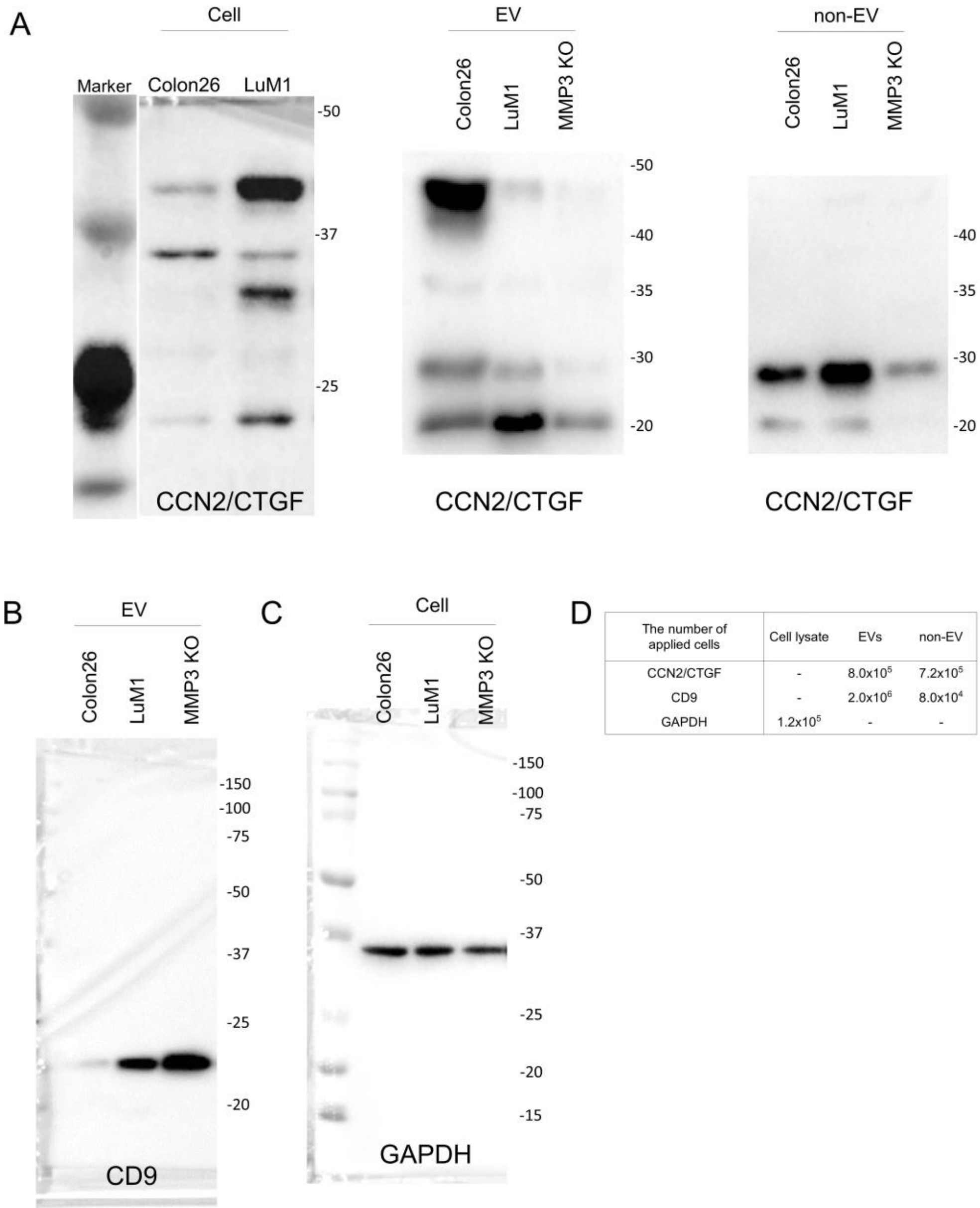

The number of applied cells	Cell lysate	EVs	non-EV
CCN2/CTGF	-	8.0×10^5	7.2×10^5
CD9	-	2.0×10^6	8.0×10^4
GAPDH	1.2×10^5	-	-

Figure S4. Full images of Western blotting of CCN2/CTGF (A), CD9 (B), and GAPDH (C), supporting Figure 5E. (D) The number of cells applied to each lane was shown.

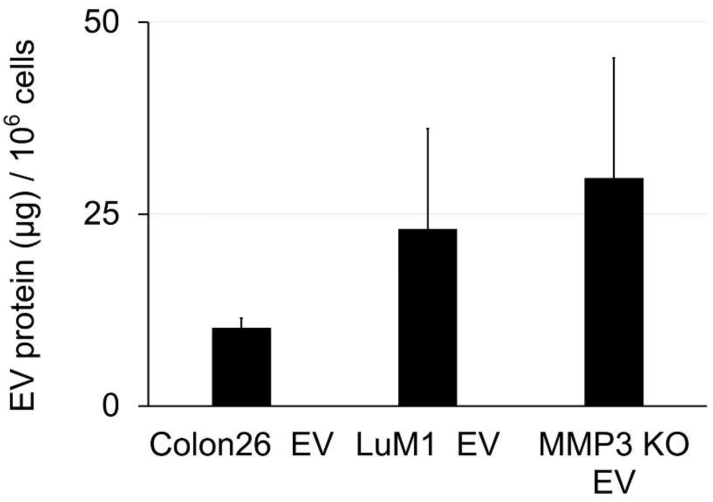

Figure S5. Protein concentrations in each EV fraction derived from Colon26, LuM1, and MMP3-KO cells.

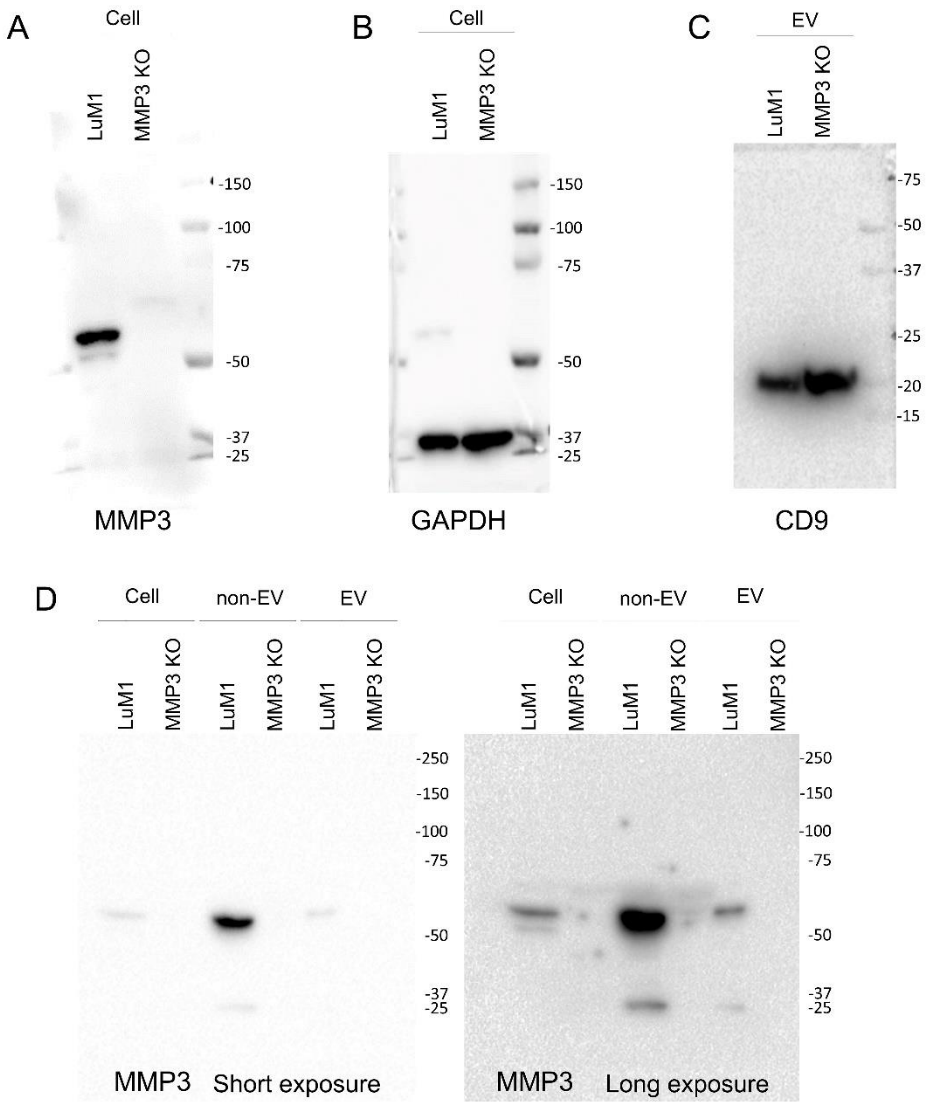

Figure S6. Full images of Western blotting, supporting Figure 3F and Figure 6A. (A, B) Western blotting of MMP3 (A) and GAPDH (B) in cell lysates. To each well, 30 μg was applied. (C, D) Western blotting of CD9 (C) and MMP3 (D), supporting Figure 6A. Protein samples prepared from 8.0 x 10^4 cells were applied to each lane.

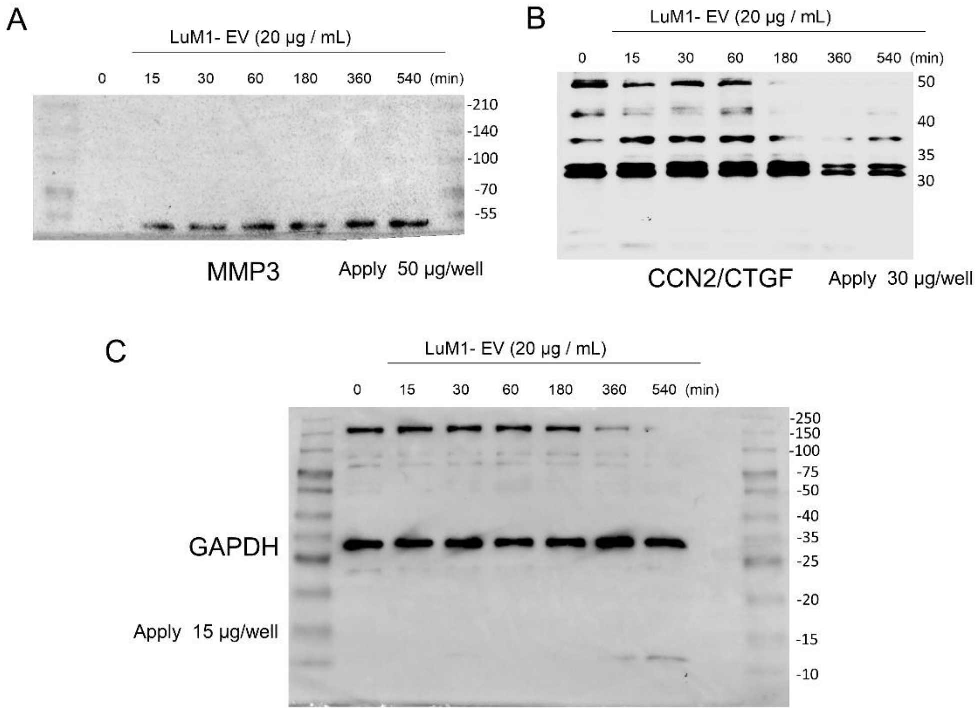

Figure S7. Full images of western blotting of MMP3 (A), CCN2/CTGF (B), and GAPDH (C), supporting Figure 7A. (A) For MMP3, 50 µg protein sample was loaded to each lane; (B) 30 µg was loaded for CCN2/CTGF; (C) 15 µg was loaded for GAPDH.
